# Glucose Modulation of the Local Conformational Flexibility and Free-Energy Basin Shape of Human and Yeast Hexokinases: A Comparative Molecular Dynamics Analysis

**DOI:** 10.3390/biology15201900

**Published:** 2026-10-21

**Authors:** Ysaías J. Alvarado, Aníbal Méndez, Lenin González-Paz, Yovani Marrero-Ponce, Félix Martinez-Rios, Joan Vera-Villalobos, Ariana Delgado, José L. Paz

**Affiliations:** 1 Centro de Biomedicina Molecular “Dr. Humberto Fernández Moran”, Instituto Venezolano de Investigaciones Científicas, Maracaibo 4001, Venezuela; alvaradoysaias@gmail.com (Y.J.A.); anibalmdzp@gmail.com (A.M.); lgonzalezpaz@gmail.com (L.G.-P.); 2 Facultad de Ingeniería, Universidad Panamericana, Mexico City 03920, Mexico; ymarrero@usfq.edu.ec (Y.M.-P.); fmartin@up.edu.mx (F.M.-R.); 3 Grupo de Medicina Molecular y Traslacional (MeM&T), Escuela de Medicina, Edificio de Especialidades Médicas, Universidad San Francisco de Quito (USFQ), Av. Interoceánica Km 12 ½ —Cumbayá, Quito 170157, Ecuador; 4 Facultad de Ciencias Naturales y Matemáticas, Escuela Superior Politécnica del Litoral, Guayaquil EC090112, Ecuador; joarvera@espol.edu.ec; 5 Facultad de Química e Ingeniería Química, Universidad Nacional Mayor de San Marcos, Departamento Académico de Química Inorgánica, Lima 15081, Peru

**Keywords:** hexokinase, free energy landscape, potential of mean force, conformational dynamics, effective volumetric compliance, molecular dynamics

## Abstract

Human hexokinases HK1 (H) and HK2 (H) are cancer drug targets, yet yeast orthologs often serve as computational surrogates. We analyzed three independent 100 ns molecular dynamics replicates of HK1 (H), HK2 (H) and their *Saccharomyces cerevisiae* homologs [HK1 (Y), HK2 (Y)] in free and glucose-bound states. Because this window samples the local free-energy basin rather than the microsecond-scale open↔closed transition, we compared how glucose modulates local flexibility and basin shape, combining potentials of mean force along the radius of gyration and backbone RMSD with basin occupancy, residence times, local well stiffness ($k_{loc}$) and volumetric compliance. In both human enzymes glucose tended to enlarge volumetric fluctuations (compliance 1.8–3.2-fold higher); HK1 (H) compacted progressively, to a replicate-dependent extent, whereas HK2 (H) showed the most reproducible effect: lower free-state basin occupancy, a 2.8-fold broader RMSD distribution and a threefold softer well ($k_{loc}$ 980 ± 145 versus 295 ± 112 *k*_B_T/nm^2^). The yeast enzymes responded weakly or oppositely: in HK1 (Y) glucose lowered backbone deviation and narrowed within-replicate fluctuations (compliance 2.5-fold lower), whereas HK2 (Y) changed minimally. In these simulations, the glucose–flexibility coupling was not conserved between isoforms or species, supporting human-specific structural ensembles, rather than yeast models, for computational inhibitor design.

## Simple Summary

Cancer cells consume unusually large amounts of sugar to fuel their rapid growth. An enzyme called hexokinase controls the very first step in which cells process this sugar, and in many tumours one of its forms is produced in excess. For this reason, scientists are trying to design drugs that block this enzyme. Because human hexokinases are large and difficult to study, researchers often use the simpler, closely related enzymes from baker’s yeast as stand-ins in computer simulations. We used computer simulations to watch how the human and yeast enzymes move and change shape when sugar binds. Repeating every simulation three times, we found indications that sugar makes the human enzymes more flexible—their overall shape tends to fluctuate more widely—whereas the yeast enzymes barely change or become slightly stiffer: the two organisms appear to respond differently, even though the enzymes look similar and share the same basic function. This suggests that yeast enzymes may not be reliable substitutes for their human counterparts when searching for new cancer drugs on the computer. Such studies may therefore be better when based on the human enzymes themselves, which could help make the search for effective cancer treatments more accurate.

## 1. Introduction

Hexokinase catalyzes the phosphorylation of glucose to glucose-6-phosphate using ATP as a phosphoryl donor, constituting the first committed and essentially irreversible step of glycolysis and a key metabolic control point in both unicellular eukaryotes and mammals [1]. In humans, four hexokinase isoforms (HK1–HK4) have been described, each exhibiting tissue-specific expression, distinct subcellular localization, and different regulatory properties. HK1 and HK2 are large (~100 kDa) bifunctional enzymes that arose through duplication and tandem fusion of an ancestral single-domain hexokinase, each comprising an N-terminal and a C-terminal domain that both resemble the yeast enzyme. The N-terminal domain of HK1 is catalytically inactive and serves a primarily regulatory function, whereas both domains of HK2 retain catalytic activity—a feature unique among mammalian hexokinases [1].

A direct consequence of this evolutionary history is that each human HK1 or HK2 enzyme [HK1 (H), HK2 (H)] is a single polypeptide chain that behaves as an intramolecular pseudo-dimer, in which the N- and C-terminal domains are each structurally homologous to the complete ~50 kDa single-domain monomer of yeast hexokinase [1,2]. The catalytically competent glucose-binding pocket of human hexokinase resides in the C-terminal domain, where crystallographic and mutagenesis studies have identified a conserved set of residues that coordinate the Glucose—Asp657, Glu708, Gln739, and Glu742, together with the catalytic Lys621—whose substitution abolishes or strongly impairs catalytic activity [2,3,4]. An equivalent constellation of paralogous residues (Ser155, Asp209, Glu260, Glu294, and the contact residue Asn208) lines a structurally analogous pocket in the N-terminal domain; this site is purely regulatory in HK1 but catalytically functional in HK2 [2,3]. This domain organization implies that a meaningful interspecies comparison must be framed as a two-domain enzyme versus a single-domain enzyme, and that the C-terminal catalytic pocket and the structurally equivalent N-terminal pocket are both acceptable reference sites for the glucose-bound conformation used in docking. Because absolute global descriptors such as the radius of gyration and the effective volumetric compliance scale with system size, the human–yeast comparison developed below is interpreted primarily through size-independent descriptors of the relative glucose response (distribution-distance metrics, multimodality, basin reweighting, and the glucose/free ratios of the local well stiffness and of the effective volumetric compliance) rather than through the absolute magnitudes themselves.

Elevated hexokinase activity is a hallmark of highly proliferative cancer cells. Most malignant tumors preferentially metabolize glucose to lactate even in the presence of oxygen, a metabolic reprogramming first described by Otto Warburg and now known as the Warburg effect [5,6]. HK2 is the predominant hexokinase isoform overexpressed in virtually all aggressive tumors [7]. Its association with the outer mitochondrial membrane via the voltage-dependent anion channel (VDAC) grants cancer cells preferential access to mitochondrially generated ATP, prevents mitochondrial permeability transition pore formation—thereby suppressing apoptosis—and facilitates the retention of glucose as glucose-6-phosphate within the cell [7]. Although HK1 is also upregulated in several malignancies, HK2 has emerged as the more attractive and selective therapeutic target given its limited expression in normal adult tissues [6,7]. These properties have motivated considerable effort toward the development of small-molecule inhibitors targeting the active sites and regulatory interfaces of HK1 and HK2.

A defining feature of hexokinase enzymology is the glucose-induced conformational change. In the substrate-free state, hexokinase adopts an open conformation in which the active site cleft is solvent-exposed; upon glucose binding, a relative rotation of the two lobes of the catalytic domain closes the cleft around the carbohydrate in a mechanism described as induced fit [8]. This structural transition, first characterized by high-resolution crystallography in yeast hexokinase [8], is associated with a measurable decrease in the radius of gyration ($R_{g}$) in solution [8] and underpins the catalytic competence of the enzyme. In broader terms, the glucose response of hexokinase may also be understood through the population shift paradigm, in which the ligand does not create new protein conformations but rather redistributes the populations of pre-existing conformational substates, selectively stabilizing those competent for catalysis [9].

The hexokinases of *Saccharomyces cerevisiae*—hereafter HK1 (Y) and HK2 (Y), corresponding to the yeast isoenzymes HXK1 (hexokinase PI) and HXK2 (hexokinase PII), respectively—are structurally homologous to the individual half-domains of human hexokinases but function as monomers of approximately 50 kDa. Their high sequence similarity with the human isoforms has led to their widespread use as structural surrogates or validation models in computational studies of hexokinase inhibition. However, yeast hexokinases fulfill regulatory functions that are absent in their human counterparts. In particular, HK2 (Y) (HXK2) plays a central role in glucose repression signaling in yeast: under high-glucose conditions, it translocates to the nucleus, where it interacts with the Mig1 transcriptional repressor and the Snf1 protein kinase to regulate the expression of numerous metabolic genes [10]. These additional, species-specific functional roles suggest that the global dynamic properties of yeast hexokinases may be shaped by selective pressures distinct from those acting on the human isoforms, potentially resulting in divergent conformational landscapes even under equivalent ligand-binding conditions.

Despite the established structural homology, a quantitative comparative analysis of how glucose binding modulates the local conformational flexibility and free-energy basins of human and yeast hexokinases—integrating thermodynamic, effective kinetic, and volumetric descriptors—has not been reported. In the present study, we analyzed three independent replicate 100 ns all-atom molecular dynamics (MD) trajectories of human HK1 and HK2 and their yeast homologs HK1 (Y) and HK2 (Y) in free and glucose-bound conditions. By reconstructing one- and two-dimensional potentials of mean force (PMF) along two global collective coordinates, the radius of gyration ($R_{g}$) and the backbone RMSD, we quantify thermodynamic basin reweighting, effective kinetic stability, local free-energy well stiffness, and volumetric flexibility in each system. Our objective is to provide a quantitative interspecies comparison of the glucose modulation of the local conformational flexibility and free-energy basin shape of each enzyme on the 100 ns timescale—that is, of the local accessible ensemble rather than the complete microsecond-scale open↔closed transition—and to assess the implications for computational drug discovery strategies targeting human hexokinases. The glucose-bound state of each enzyme is defined as the occupancy of all its crystallographically established glucose-binding sites (two in the human enzymes, one in the yeast enzymes); catalysis, binding affinity and the effects of co-ligands such as glucose 6-phosphate, phosphate or ADP lie outside the scope of this work.

## 2. Materials and Methods

### 2.1. Simulated Systems and Conditions

We analyzed 100 ns MD trajectories of human HK1 and HK2 and their yeast homologs (*Saccharomyces cerevisiae*; HK1 (Y) and HK2 (Y)), in the ligand-free state and in complex with glucose (+Glu). The ligand-free state is referred to as “free” rather than “apo” throughout, because three of the four ligand-free systems were obtained by removing the crystallographic ligands from holo structures rather than from genuinely apo crystal forms. Initial structures were obtained from the Protein Data Bank: 1CZA (human HK1), 2NZT (human HK2), 3B8A (HK1 (Y)), and 1IG8 (HK2 (Y)). Of these, 1CZA (crystallized with glucose, glucose-6-phosphate and ADP), 2NZT (glucose and glucose-6-phosphate) and 3B8A (glucose; closed form) are ligand-bound structures; after removal of all their ligands, only glucose was re-docked to construct the glucose-bound systems, so that the free/+Glu contrast reflects the effect of glucose alone. In contrast, 1IG8 (HK2 (Y)) is a ligand-free, open-form structure. In the glucose-bound systems, glucose occupied every crystallographically established glucose-binding site of the enzyme: in the human enzymes, the C-terminal catalytic site and the N-terminal site, catalytic in HK2 [3] and, in HK1, a non-catalytic “vestigial active site” that binds glucose in the crystal structures 1HKB, 1HKC, 1CZA and 1DGK [2,11,12]; in the yeast enzymes, glucose occupied the single sugar-binding site. For HK2 (Y), the glucose-bound system was built in a closed conformation: as no closed crystal structure of the PII isoform is available, the two domains of 1IG8 were placed in the closed arrangement by rigid-body superposition onto the closed, glucose-bound structure of the paralogous PI isoform (PDB 3B8A), and glucose was then placed into this closed model, at the site equivalent to the sugar-binding site of PI, with the same blind docking protocol used for all systems (Section 2.7). For each system, a single polypeptide chain was retained as the simulated unit: in the human enzymes this corresponds to the complete ~100 kDa two-domain chain (N- and C-terminal domains), whereas in the yeast enzymes it corresponds to the ~50 kDa single-domain monomer. Consequently, the larger absolute radius of gyration of the human systems reflects their two-domain architecture rather than an oligomeric assembly. These structures differ in sequence length, unresolved regions, and presence of mutations or crystallographic ligands. No homology modeling, loop reconstruction or AlphaFold-based completion was performed. The deposited structures were treated as initial conditions from which each system relaxes, and all analyses are based on the dynamically sampled conformational ensembles rather than on individual crystallographic conformations. Initial structural differences are acknowledged as a potential source of bias. In particular, unresolved segments may contribute to the absolute $R_{g}$ and volumetric fluctuations from which the glucose/free ratios are obtained. Because the free and glucose-bound systems of each enzyme were built from the same deposited coordinates, the glucose response of each enzyme is measured between ensembles with identical unresolved regions. Throughout this work, residue numbers given in the main text and figure legends follow the crystallographic numbering of the deposited structures, whereas residue numbers reported in the Supplementary Material follow the simulation-topology numbering, which corresponds directly to the residue indices of the deposited trajectory files.

Molecular dynamics simulations were performed using GROMACS (version 2024.3) [13] with the AMBER ff14SB all-atom force field [14] for the protein and the GLYCAM06j-1 carbohydrate force field [15] for glucose, modeled as β-d-glucopyranose (GLYCAM residues 0 GB and ROH), the predominant anomer in aqueous solution and the one bound in the HK1 crystal structures 1HKB and 1HKC [2,11]. Each system was assembled in LEaP (AmberTools26), solvated in a cubic periodic box with TIP3P water molecules and neutralized with Na^+^ and Cl^−^ counter-ions to achieve physiological ionic strength (0.15 M NaCl). The resulting AMBER topology stores the 1–4 scale factors per dihedral (SCEE = 1.2 and SCNB = 2.0 for the protein; SCEE = SCNB = 1.0 for glucose, as required by GLYCAM06) and was converted to GROMACS with ACPYPE (version 2026.9.3), which writes each 1–4 pair explicitly with its own scale factors [16] instead of applying a single global fudgeLJ/fudgeQQ pair. Because glucose is not covalently bound to the protein, protein–glucose interactions involve no 1–4 pairs and were computed with the full Lennard-Jones and particle-mesh Ewald terms. Energy minimization was carried out using the steepest-descent algorithm until convergence. Thermal equilibration was performed in the NVT ensemble for 1000 ps using the velocity-rescaling thermostat [17] at 300 K, followed by pressure equilibration in the NPT ensemble for 1000 ps with the Parrinello–Rahman barostat [18] at 1 bar. Long-range electrostatic interactions were computed using the Particle Mesh Ewald method [19], and all bonds involving hydrogen atoms were constrained with the LINCS (Linear Constraint Solver) algorithm [20]. Production runs were integrated with a 2 fs time step in three independent 100 ns replicates for each system and condition, with coordinates saved every 0.01 ns (10,001 frames per trajectory). The representative (dynamized) structure for each system was defined as the centroid of the most populated conformational cluster obtained by GROMOS clustering of the backbone RMSD over the three pooled replicate trajectories (after discarding the equilibration period)—that is, the dominant free-energy basin rather than an arbitrary single frame; the convergence of this basin across the three replicates was verified. Structural observables—root-mean-square deviation (RMSD), root-mean-square fluctuation (RMSF), and $R_{g}$—were computed from the production trajectories.

### 2.2. Conformational Descriptors and Statistical Analysis

For each frame of the trajectories, two global collective coordinates were extracted: (i) the $R_{g}$, as a measure of global compaction or expansion, and (ii) the RMSD with respect to the reference structure of each simulation, as an indicator of global structural deviation.

The marginal distribution of $R_{g}$, $P\left(R_{g}\right)$, was estimated for each system in free and +Glu conditions by normalized histograms and kernel density estimation (KDE) [21]:

(1) $$P\left(R_{g}\right)=\frac{1}{nh}\sum_{i=1}^{n}K\;\left(\frac{R_{g}-R_{g,i}}{h}\right)$$

where *n* is the number of samples, *h* is the bandwidth, and K is a Gaussian kernel. The KDE was evaluated with a Gaussian kernel and an automatically selected (Scott’s-rule) bandwidth, and the accompanying normalized histograms used Freedman–Diaconis bin widths, so that no free smoothing parameter was tuned by hand. Multimodality was quantified by counting the local maxima of the KDE, retaining only peaks whose relative prominence exceeded 0.08 of the total density range. The glucose-induced shift in the distribution was quantified as:

(2) $${∆R}_{g}=median\left(R_{g,+Glu}\right)-median\left(R_{g,free}\right)$$

Unless otherwise stated, scalar descriptors were computed for each replicate and are reported as the between-replicate mean ± SD (*n* = 3), whereas the $R_{g}$ and RMSD distributions and the free-energy profiles derived from them pool the frames of the three replicates. In addition to the pooled distributions, each replicate was characterized by the median, the interquartile range (IQR) and the linear drift (least-squares slope versus time) of $R_{g}$ and of the backbone RMSD. The reproducibility of the glucose effect was assessed by computing, for each replicate pair (R-1 (free) versus R-1 (+ Glu), and so on), the difference Δ*X* = *X*(+Glu) − *X*(free) of the median $R_{g}$, the time-averaged $R_{g}$ and the median RMSD, and by recording whether Δ*X* had the same sign in the three pairs (sign consistency). Because the free and glucose-bound replicates are independent simulations, this pairing is nominal. With *n* = 3, a glucose effect was regarded as robust only when the replicate ranges of the two conditions did not overlap, and as indicative when its sign was consistent across the three replicate pairs or between the between-replicate and pooled estimates.

### 2.3. Potentials of Mean Force (PMF)

One-dimensional PMFs were reconstructed for each collective coordinate (the radius of gyration and the backbone RMSD) by Boltzmann inversion of its kernel-density estimate. Written explicitly for the radius of gyration, $F\left(R_{g}\right)$ is given by [21,22]:

(3) $$F\left(R_{g}\right)=-k_{B}T\ln\left[P\left(R_{g}\right)\right]+C$$

where $P\left(R_{g}\right)$ is the kernel-density estimate of the corresponding distribution, k*B* is the Boltzmann constant, *T* is the simulation temperature, and C is a constant setting the global minimum of F to zero. The backbone-RMSD profile, F(RMSD), was obtained in exactly the same way. Each profile was evaluated on a regular grid of 300 points spanning the 0.2nd–99.8th percentiles of the sampled coordinate; grid points whose kernel density fell below 0.1% of its maximum were treated as unsampled and left as gaps, so that sparsely populated regions do not appear as spurious high-energy barriers. Because these profiles derive from unbiased trajectories—the frames of the three replicates being pooled for each condition, so that between-replicate differences in the sampled basin contribute to the width of the profiles—they are interpreted as effective, relative free-energy landscapes for comparison between equivalent systems rather than as fully converged potentials of mean force [22]. They were used to locate the dominant minima and to estimate the effective width of the free-energy wells.

Two-dimensional PMFs $F\left(R_{g},\;RMSD\right)$ were constructed by Boltzmann inversion of the joint probability $P\left(Rg,RMSD\right)$ [23]:

(4) $$F\left(R_{g},\;RMSD\right)=-k_{B}T\ln\left[P\left(Rg,RMSD\right)\right]+C$$

The joint probability was estimated from a two-dimensional histogram of the paired ($R_{g}$, RMSD) values, obtained by matching the two time series frame-by-frame to their nearest common time point. These 2D landscapes allow identifying conformational basins, interconversion routes, and glucose-induced topological changes in the free-energy landscape [23].

### 2.4. Local Free-Energy Well Stiffness

Around the principal minimum of $F\left(R_{g}\right)$, a local quadratic fit was performed over a symmetric window spanning approximately ±12% of the sampled range of the coordinate, to characterize the curvature of the energy well [24]:

(5) $$F\left(R_{g}\right)\approx a{\left(R_{g}-R_{g,0}\right)}^{2}+b\left(R_{g}-R_{g,0}\right)+c$$

where $R_{g,0}$ is the minimum position. The local stiffness was defined as:

(6) $$k_{loc}=2\cdot a\;(in\;units\;of\;k_{B}\cdot T/{nm}^{2})$$

High $k_{loc}$ values indicate a narrow minimum (low local fluctuation), while low values indicate a flatter well (greater conformational permissiveness) [24]. Uncertainties were estimated by non-parametric bootstrap resampling (*n* = 400 resamples); the reported 95% confidence intervals correspond to the 2.5th and 97.5th percentiles of the bootstrap distribution. $k_{loc}$ of each replicate was taken as the mean of its bootstrap distribution; for comparison, it was also computed from the pooled frames of the three replicates, for which the bootstrap 95% confidence interval is reported. Because this frame-level bootstrap does not account for the temporal autocorrelation of the trajectories, these intervals are narrower than the run-to-run dispersion, and the between-replicate SD is used as the primary measure of uncertainty.

### 2.5. Analysis of Conformational Basins

Conformational basins were defined from the one-dimensional PMF $F\left(R_{g}\right)$ [25]. Local minima were identified as dominant metastable states, and the intermediate local maxima were used as basin boundaries. In practice, the minima of $F\left(R_{g}\right)$ were located as the maxima of the kernel-density estimate whose prominence exceeded 0.08 of the density range (the criterion used to count the KDE peaks), and each boundary was placed at the density minimum (maximum of *F*) between the dominant minimum and the neighbouring one; in addition, the basin was restricted to the contiguous region around the dominant minimum in which *F* does not exceed 2 *k*_B_*T*, so that its limits are set by the free-energy profile itself when no neighbouring minimum exists. The dominant basin was defined on the PMF of the pooled free-state frames of each enzyme and applied to both conditions, so that its occupancy in the +Glu ensemble measures the fraction of glucose-bound frames that remain within the basin of the free enzyme. Basin occupancy was calculated as the fraction of trajectory frames whose $R_{g}$ value falls within the limits of each basin, providing a direct estimate of the relative thermodynamic weight of each substate.

To incorporate an effective kinetic dimension, apparent residence times (ART) were estimated by identifying continuous temporal segments in which the trajectory remains within the same basin and calculating their mean duration [26]. ART was computed on a 0.1 ns grid (every tenth frame of the 0.01 ns trajectories), so that recrossings of the basin boundaries shorter than 0.1 ns are not resolved; ART at the native 0.01 ns sampling is given in Table S5 for reference. Because ART cannot exceed the 100 ns trajectory length, it is bounded in replicates with few exits from the basin. This parameter is used as a comparative measure of the relative kinetic stability of dominant states under each condition, rather than a rigorous description of microscopic transition kinetics. A decrease in ART suggests greater conformational lability; an increase indicates greater temporal persistence of the corresponding state [27].

To quantify the glucose-induced changes in the conformational ensemble at the level of whole distributions, we computed two complementary distribution distance metrics between the free and +Glu conditions for both $R_{g}$ and RMSD: the Jensen–Shannon divergence (JSD) [28], which is symmetric, bounded, and sensitive to global probability redistributions, and the Bhattacharyya distance (BD) [29], which measures the degree of overlap between distributions. Both were computed from kernel-density estimates evaluated on a common grid of 300 points spanning the 0.2nd–99.8th percentiles of the combined free and +Glu data, with the JSD expressed in base 2 (bounded between 0 and 1); histograms with Freedman–Diaconis bins gave pooled values differing by at most 0.014 (JSD) and 0.031 (BD). To distinguish a glucose effect from run-to-run variability, each distance was computed (i) between the pooled free-state and +Glu ensembles, (ii) for the nine cross pairs of individual free and +Glu replicates, and (iii) for the six pairs of replicates of the same condition, which provide a between-replicate baseline; a ratio of cross-pair to baseline distance close to 1 indicates that the free/+Glu difference does not exceed the variability between independent runs. Together, these metrics quantify the ligand impact on each system relative to its run-to-run variability.

### 2.6. Effective Volumetric Compliance

The effective cavity volume, $V_{c}$, of the protein in solution was estimated from $R_{g}$ using a spherical model with shape factor ${R=\alpha R}_{g}$, $\alpha =\sqrt{5/3}$, so that $V_{c}\left(t\right)=\frac{4}{3}\pi \alpha^{3}{\left[R_{g}\left(t\right)\right]}^{3}$. From the fluctuations of $V_{c}\left(t\right)$ in the NPT ensemble (300 K, 1 bar) employing the Parrinello–Rahman barostat in the MD simulation, the effective volumetric compliance of the protein was estimated as:

(7) $$\kappa_{T}^{eff}=\frac{\langle V_{c}^{2}\rangle -{\langle V_{c}\rangle }^{2}}{k_{B}T\langle V_{c}\rangle }$$

Although this quantity is formally analogous to an isothermal compressibility, it is not a thermodynamic compressibility: it is derived from the fluctuations of a coarse geometric cavity volume rather than from the true volume fluctuations of the solvated protein, and we therefore refer to it as an effective volumetric compliance. This descriptor quantifies the global conformational volumetric flexibility of the ensemble and is used for relative comparisons between systems and conditions. The 95% confidence intervals reported for this descriptor were obtained by a moving-block bootstrap (1000 replicates) that resamples contiguous trajectory segments, with a block length of the order of the cube root of the number of frames, so as to preserve the temporal autocorrelation of the cavity-volume time series. $\kappa_{T}^{eff}$ was also computed from the pooled frames of the three replicates. The values of $\kappa_{T}^{eff}$ obtained here are not directly comparable to experimental compressibilities reported for globular proteins in solution (~10–20 Mbar^−1^) [30,31], since the effective geometric cavity amplifies global conformational fluctuations by several orders of magnitude. Accordingly, $\kappa_{T}^{eff}$ is interpreted strictly as a relative volumetric flexibility descriptor under the same methodological scheme. Because the cavity volume is a deterministic function of the radius of gyration, this descriptor—like the KDE, the one-dimensional PMF along $R_{g}$, and the local well stiffness—summarizes the same $R_{g}$ distribution rather than providing information independent of it; the backbone RMSD (used in the two-dimensional PMF and in the RMSD-based separation metrics) provides the complementary, independent structural coordinate.

### 2.7. Molecular Docking and Binding-Site Validation

The glucose-bound systems used as starting structures for the molecular dynamics simulations were generated by molecular docking of D-glucose (PubChem CID 5793), modeled in the simulations as the β-D-glucopyranose anomer, into each hexokinase structure. Blind molecular docking was then performed with CB-Dock3 [32,33], a cavity-detection-guided protein–ligand docking protocol that identifies candidate binding cavities across the whole protein surface and ranks ligand poses without requiring a predefined search box. This blind strategy was adopted so that the candidate binding cavities emerged from the structural features of each protein rather than from a predefined search box.

For each docked complex, the protein–ligand interaction profile was inspected and compared against the active-site residues reported in the crystallographic literature for the corresponding enzyme. For the human enzymes, the conserved C-terminal catalytic pocket (Asp657, Glu708, Gln739, Glu742, Lys621) and the structurally equivalent N-terminal glucose-binding pocket were both considered as reference sites; for the yeast enzymes, the single-domain sugar-binding site of yeast hexokinase was used as the reference. In each reference pocket, the highest-scoring CB-Dock3 pose was retained. The selected poses were subsequently re-scored with the energetic functions implemented in Molegro [34]—MolDock, PLANTS, and ReRank—to provide a comparative, internally consistent estimate of the protein–ligand binding energetics under a single scoring scheme. Because experimentally resolved protein–ligand complexes defining the interacting residues are not available for all systems, these re-scoring values are interpreted as relative descriptors of pose quality within the present methodological framework rather than as absolute binding free energies. The validated glucose-bound poses were then used as the initial conditions for the +Glu trajectories described in Section 2.1.

### 2.8. Ligand Persistence Analysis and Sampling Strategy

To verify that glucose remained associated with its binding cleft throughout the trajectories, the persistence of the ligand in each pocket was quantified from the production runs. After least-squares superposition of each frame onto the protein Cα atoms, the protein-aligned RMSD of the glucose heavy atoms relative to the equilibrated pose was computed as a measure of ligand displacement, and the fraction of frames with ligand RMSD below 0.35 nm was used as a residence metric. In addition, per-residue contact occupancy was defined as the fraction of frames in which any atom of a given pocket residue lay within 0.35 nm of the ligand, using the binding-site residues automatically detected within 4.5 Å of glucose in the equilibrated structure. For the two-domain human enzymes, in which glucose occupied both the N- and C-terminal sites, each bound glucose was analyzed independently in its own pocket. Ligand displacement and per-residue contact occupancies were computed with the MDAnalysis library [35].

The use of three independent 100 ns replicates per system and condition was chosen because the objective of the present study was not to estimate ligand binding affinities or converged thermodynamic properties—quantities for which the glucose–hexokinase interaction has already been extensively characterized experimentally and structurally [2,3,8]—but rather to perform a comparative characterization of the glucose modulation of the local conformational flexibility and free-energy basin shape of each enzyme under a defined ligand-bound state. This simulation protocol is consistent with numerous published molecular-dynamics studies using structural descriptors such as backbone RMSD, RMSF, $R_{g}$, and intermolecular interaction persistence [36,37]. Accordingly, all systems were simulated under identical conditions, and subsequent comparisons were performed within a consistent computational framework using relative structural descriptors rather than quantitative estimates of absolute free-energy values.

## 3. Results and Discussion

**Figure 1:**
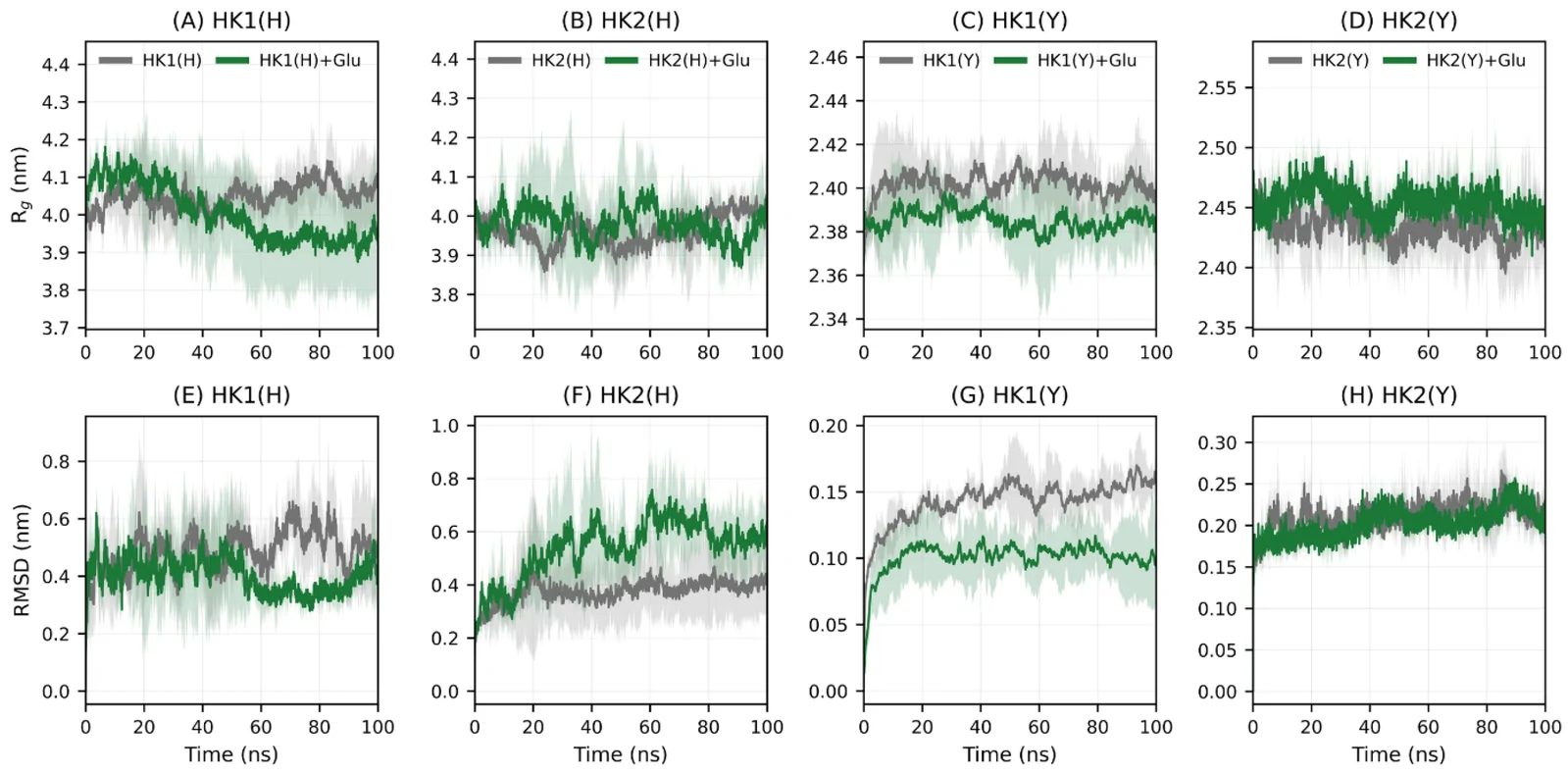
Backbone stability over the 100 ns production trajectories of the four hexokinases in the free and glucose-bound (+Glu) states. Top row (**A**–**D**): radius of gyration ($R_{g}$); bottom row (**E**–**H**): backbone RMSD. Columns, from left to right: HK1 (H), HK2 (H), HK1 (Y) and HK2 (Y). Each panel shows the mean (solid line) ± SD (shaded band) across three independent replicates for the free enzyme (grey) and the glucose-bound enzyme (forest green). Glucose was described with GLYCAM06, and the glucose-bound HK2 (Y) trajectories start from the closed model (Section 2.1). H: human isoform; Y: yeast (*Saccharomyces cerevisiae*) isoform. Per-replicate traces for all eight system–condition combinations are shown in Figure S1.

In all four enzymes, glucose was docked in the conserved glucose-binding site of each inter-domain cleft (Section 2.7), in agreement with the sugar-binding architecture reported for hexokinase. After an initial relaxation of ~20 ns, the radius of gyration and backbone RMSD of most systems fluctuated around stable plateaus (Figure 1; Figure S1); the one exception—the progressive compaction of glucose-bound HK1 (H)—is quantified below.

Figure 1 summarizes the backbone stability of the eight system–condition combinations as the mean ± SD over the three independent replicates, so that the shaded envelope reflects run-to-run dispersion rather than a single trajectory; the per-replicate traces are shown in Figure S1 and the per-replicate descriptors in Table S1. The replicate-averaged median backbone RMSD values of the human isoforms were 0.496 ± 0.101 (free) and 0.387 ± 0.072 nm (+Glu) for HK1 (H) and 0.376 ± 0.132 (free) and 0.534 ± 0.131 nm (+Glu) for HK2 (H) (Table 1). Glucose lowered the RMSD of HK1 (H) in all three replicate pairs (ΔRMSD = −0.109 ± 0.137 nm), whereas in HK2 (H) it raised the median RMSD in two of the three pairs (+0.157 ± 0.243 nm) and, more robustly, broadened the RMSD distribution 2.8-fold (interquartile range 0.065 ± 0.028 versus 0.180 ± 0.058 nm, with non-overlapping replicate ranges), consistent with an induced-fit rearrangement of the bilobal cleft rather than a simple rigidification. The $R_{g}$ of free HK1 (H) drifted slightly upward in all three replicates (slopes +0.14 to +0.81 × 10^−3^ nm ns^−1^), whereas that of glucose-bound HK1 (H) decreased in all three (−0.81 to −4.04 × 10^−3^ nm ns^−1^); the glucose-bound HK1 (H) ensemble therefore had not reached a stationary $R_{g}$ within 100 ns, and its distribution-based descriptors include this progressive compaction. The two single-domain yeast enzymes were the most rigid and stationary systems. HK2 (Y) (median RMSD 0.211 ± 0.017 versus 0.201 ± 0.008 nm; all slopes ≤0.71 × 10^−3^ nm ns^−1^ in absolute value) was nearly indistinguishable between conditions. HK1 (Y), rebuilt from the closed, glucose-bound structure 3B8A (Section 2.1), showed the smallest backbone deviation of all systems (free-state median RMSD 0.143 ± 0.004 nm, with a residual drift of +0.31 to +0.47 × 10^−3^ nm ns^−1^) and a flat $R_{g}$ (all slopes ≤ 0.24 × 10^−3^ nm ns^−1^ in absolute value); with glucose its median RMSD decreased in all three replicate pairs, with non-overlapping replicate ranges (0.102 ± 0.022 nm; ΔRMSD = −0.040 ± 0.019 nm; Table S2), that is, the glucose-bound enzyme remained closer to its glucose-bound starting conformation. The per-residue RMSF profiles (Figure S1) placed the mobile segments at the same positions in the three replicates; apart from the chain termini, the largest fluctuations were confined to a segment near residue 580 of glucose-bound HK2 (H) (≈0.7 nm), whereas both yeast enzymes remained below ≈0.3 nm. Across systems, the sign of the glucose effect on $R_{g}$ and RMSD was reproducible in all three replicate pairs for HK1 (H) (time-averaged $R_{g}$ and median RMSD; Table S2) and HK1 (Y) (median $R_{g}$ and RMSD), so that, at *n* = 3, only effects exceeding the run-to-run dispersion are interpreted in the following sections.

**Figure 2:**
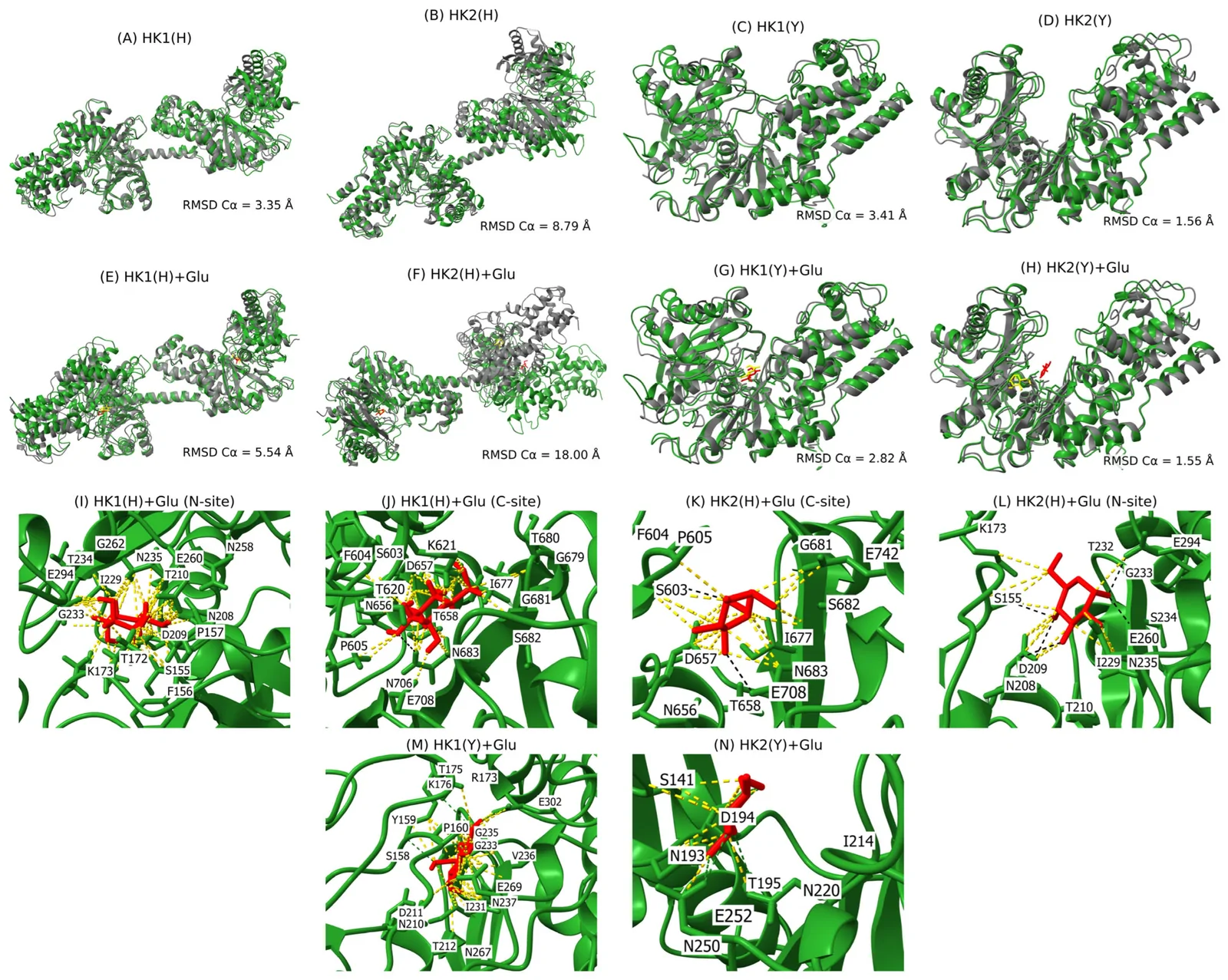
Crystal versus dynamized (MD) conformations of the four hexokinases. In each superposition the experimental or docked initial structure is shown in grey and the dynamized representative in forest green (centroid of the most populated conformational basin, pooled over the three 100-ns replicates). (**A**–**D**) Free enzymes, crystal vs. MD: (**A**) human HK1 [HK1 (H)], (**B**) human HK2 [HK2 (H)], (**C**) yeast HK1 [HK1 (Y)], (**D**) yeast HK2 [HK2 (Y)]. (**E**–**H**) Glucose-bound complexes in a common orientation, initial docking pose vs. MD, in the same order (for HK2 (Y), the initial structure is the closed model described in Section 2.1). (**I**–**N**) Close-ups of the glucose pocket in the dynamized complex, showing the MD glucose pose (red sticks) with the contacting residues (labelled), hydrogen bonds (black dashed lines) and other contacts (yellow dashed lines); because the two human enzymes bind glucose in both halves, their N-terminal (regulatory in HK1, catalytic in HK2) and C-terminal catalytic pockets are shown separately—(**I**) HK1 (H) N-domain, (**J**) HK1 (H) C-domain, (**K**) HK2 (H) C-domain, (**L**) HK2 (H) N-domain—whereas (**M**) HK1 (Y) and (**N**) HK2 (Y) are single-domain. The value in each superposition panel is the Cα RMSD between the initial and the dynamized structure (all matched Cα atoms, common orientation). The human enzymes (**A**,**B**,**E**,**F**) retain the bilobal two-domain architecture, whereas the yeast enzymes (**C**,**D**,**G**,**H**) are single-domain.

The dynamized representative of each system—the centroid of its most populated conformational cluster pooled over the three replicates (Section 2.1)—is shown in Figure 2 superposed on its starting structure, for the free enzymes and for the glucose-bound complexes of human HK1 and HK2 and HK1 (Y) and HK2 (Y) that underlie the conformational-landscape analysis developed in the following sections. The dynamized representatives preserved the global fold of all four hexokinases relative to their experimental or docked initial structures. The magnitude of the initial-to-MD Cα deviation nevertheless differed markedly between systems (values indicated in each panel of Figure 2): it was smallest for HK2 (Y), both free and glucose-bound (1.56 and 1.55 Å), followed by HK1 (Y) (3.41 and 2.82 Å) and free HK1 (H) (3.35 Å), and largest for HK2 (H) (8.79 Å free; 18.00 Å +Glu) and glucose-bound HK1 (H) (5.54 Å). In the two-domain human enzymes, the N- and C-terminal domains, joined by a connecting segment, reorient relative to each other during the simulations and therefore cannot be superposed simultaneously, so that the superposition aligns a single domain and the Cα RMSD is dominated by the rigid-body displacement of the other. This inter-domain reorientation is consistent with a mode documented crystallographically for HK1, in which the N-terminal half rotates as a rigid body about the N-terminus of the transition helix that connects the two halves, its orientation depending on the ligand (phosphate or glucose 6-phosphate) bound with glucose at the vestigial active site of this half [12]. In glucose-bound HK1 (H) (Figure 2E), this rigid-body displacement of one half relative to the other is consistent with the progressive compaction of this isoform arising from a hinge-like closure of the two halves. These deviations reflect rigid-body motions of domains and lobes and loop repacking rather than global unfolding on the 100 ns timescale, consistent with the local-flexibility interpretation adopted throughout this work. Importantly, glucose binding did not rigidify the human enzymes globally (Figure 1); instead, the sugar remained anchored within the inter-domain cleft by a conserved and reproducible set of hydrogen-bonded contacts (Figure 2I–N), while the surrounding pocket retained appreciable conformational freedom—the structural signature that motivates the free-energy-basin analysis developed below.

The three replicates were also compared at the level of the whole dynamized structure (Figure S2). Mutual superposition of the three per-replicate representative structures gave inter-replicate Cα RMSD values of 3.65–4.77 Å for the human enzymes in both conditions and of 2.12 and 1.59 Å (HK1 (Y)) and 1.43 and 1.36 Å (HK2 (Y)) for the free and glucose-bound yeast enzymes, indicating convergent sampling in all four systems.

Because all subsequent analyses assume that glucose remains within its binding cleft, the persistence of the ligand was quantified over the production trajectories of the three replicates (Section 2.8; Table S4 and Figure S3). In all six analyzed sites the glucose molecule remained tightly bound, with a low and stable protein-aligned RMSD (median 0.05–0.16 nm; 95th percentile 0.10–0.37 nm) and residence within the cavity (ligand RMSD < 0.35 nm) in 98.5–99.9% of frames. The most tightly retained ligands were those of the yeast enzymes: in HK1 (Y) (median RMSD 0.05 nm; 95th percentile 0.10 nm; 99.9% residence; mean contact occupancy 0.81) glucose remained in contact with Ser158, Thr175, Lys176, Asp211 and Asn237, the residues that coordinate it in the 3B8A crystal structure, and in HK2 (Y) the corresponding values were 0.08 nm, 0.14 nm and 99.9%. In the human enzymes, glucose persisted in both the N- and C-terminal pockets (98.5–99.6% residence). Overall, this confirms that the glucose-occupied state assumed throughout the conformational-landscape analysis is maintained during the simulations, and that the divergent responses described below occur under a genuinely and persistently ligand-bound condition.

Within each cavity, glucose engaged the conserved sugar-binding residues, as quantified by the per-residue contact occupancy averaged over the three replicates (Figure S3; Table S4). The mean pocket contact occupancy was 0.75 (N-site) and 0.68 (C-site) in HK1 (H) and 0.65 (N-site) and 0.71 (C-site) in HK2 (H), and the catalytic-paralogue aspartates Asp209 (N-pocket) and Asp657 (C-pocket) were among the persistent contacts in both isoforms (Figure S3). The N-terminal pocket—regulatory in HK1 but catalytically competent in HK2—is a bona fide physiological glucose-binding site in the deposited human structures [2,3]; consistently, Asp209 corresponds to the residue reported to hydrogen-bond the 4-OH and 6-OH of glucose in the N-domain of HK2 [3]. In accordance with the definition of the glucose-bound state (Section 2.1), glucose occupied both pockets of the human enzymes; in the HK1 crystal structures, the N-terminal pocket binds glucose together with glucose 6-phosphate or phosphate [12], and in the glucose/phosphate complex both ligands stabilize this half in a closed conformation [11]. The glucose responses reported for the human enzymes therefore correspond to the occupancy of both sites; how they partition between the catalytic and the non-catalytic site of HK1 is not resolved by the present simulations and would require single-site (C-terminal-only) simulations. In particular, the progressive compaction of HK1 (H), and the larger volumetric compliance that accompanies it, may depend on the occupancy of its N-terminal site.

Quantifying this contact pattern across the three independent replicates (mean ± SD; Figure S3) resolved the glucose-binding fingerprint of each pocket and confirmed its reproducibility. In the human enzymes, the N- and C-terminal pockets showed distinct, domain-specific fingerprints (Figure 2I–L). In HK1 (H) the regulatory N-pocket was dominated by the charged cluster Glu260, Glu294, Lys173 and Asp209 together with Thr172, whereas the catalytic C-pocket was anchored chiefly by Glu708, Pro605 and the catalytic aspartate Asp657; in HK2 (H) the same conserved anchors recurred (Glu260, Asp209 and Ser155 in the N-pocket; Asp657 and Glu742 in the C-pocket). The catalytic aspartate (Asp657) and the acidic Glu708/Glu742 pair—which coordinate the glucose hydroxyls and constitute the conserved catalytic/sugar-binding core of the hexokinase fold [2,3]—were consistently among the highest-occupancy, lowest-variance contacts, providing an internal, replicate-reproducible validation of the docked poses. In the single-domain yeast enzymes the pocket was defined by Ser158, Asp211, Thr175 and Lys176, together with Arg173, Pro160 and Asn237, in HK1 (Y) (mean pocket occupancy 0.81) and by Asn210 and Asp211 in HK2 (Y) (mean pocket occupancy 0.60). Across all three replicates the per-residue occupancies were graded rather than saturating (Figure S3; Table S4): a small core of anchors (e.g., the pocket glutamates in the human enzymes and Ser158 in HK1 (Y)) was occupied in the majority of frames in every replicate, while more peripheral polar and non-polar contacts fluctuated between replicates, consistent with a cavity that grips the sugar through a conserved anchor set while retaining appreciable local plasticity.

Within this framework, the representative structure of the HK1 (H) regulatory N-pocket also shows a donor–donor contact between glucose and Thr234 (Figure 2I), consistent with a glucose-binding geometry that, having lost catalytic competence, is not optimized for an ideal hydrogen-bond network. However, Thr234 is not among the persistent contacts identified across the three replicates (Figure S3; Table S4), so this local feature is not interpreted as a structural correlate of the conformational-landscape descriptors developed in the following sections.

**Figure 3:**
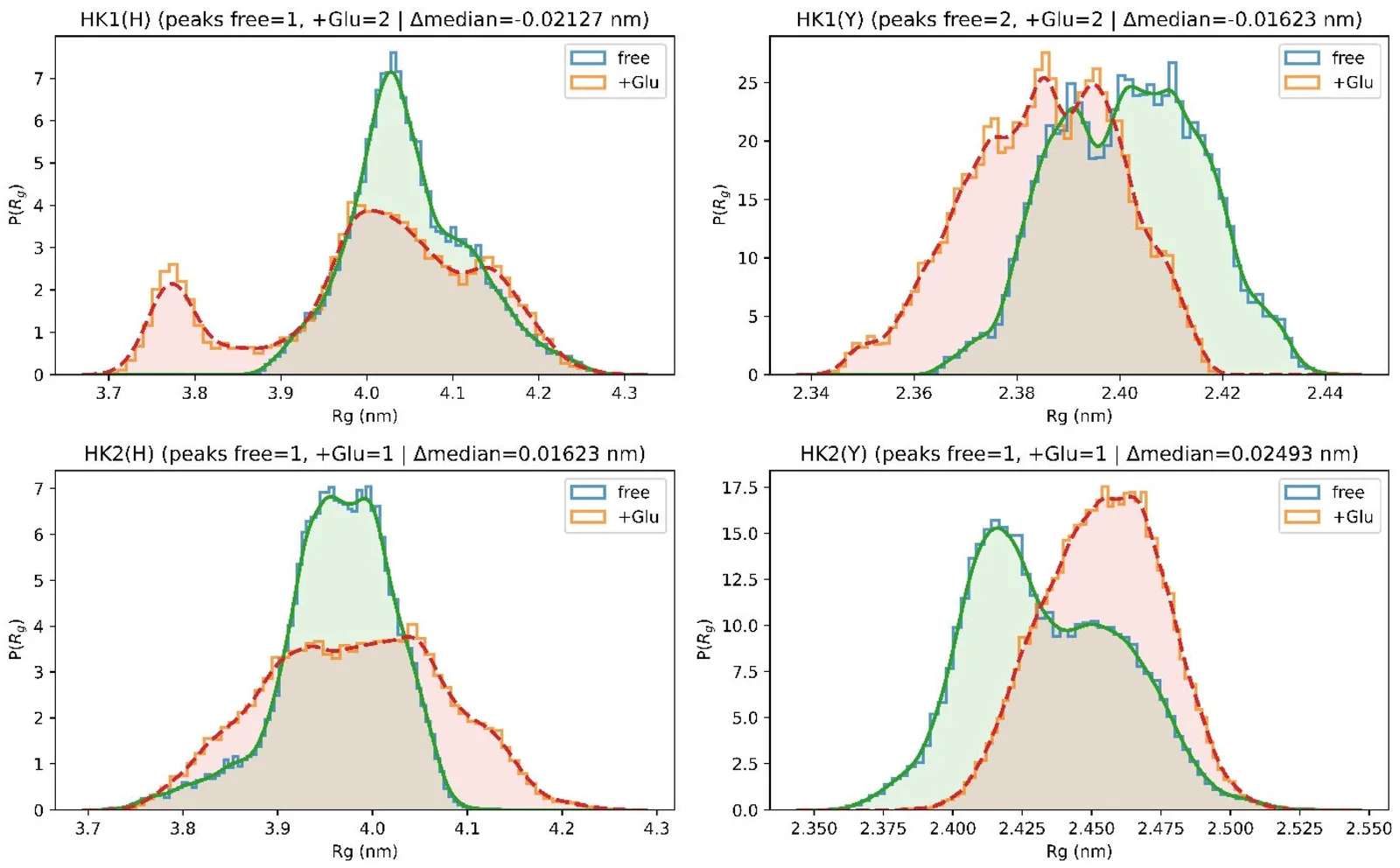
Radius-of-gyration distributions, $P(R_{g})$, for the four hexokinases in the free and glucose-bound (+Glu) states, computed from the pooled frames of the three replicates. Normalized histograms (Freedman–Diaconis bins; free: blue; +Glu: orange) are overlaid with kernel-density estimates (free: green solid; +Glu: red dashed). Panel titles report the number of KDE peaks per condition and the glucose-induced median shift, Δmedian = median($R_{g}$,+Glu) − median($R_{g}$,free).

The $R_{g}$ distributions (Figure 3; Table 1) show that the glucose response of the human enzymes consists mainly of a change in the width and position of the ensemble rather than a uniform shift. In human HK1, glucose lowered the replicate-averaged median $R_{g}$ from 4.052 ± 0.058 to 3.990 ± 0.139 nm and approximately doubled its interquartile range (0.068 ± 0.026 versus 0.142 ± 0.071 nm). The time-averaged $R_{g}$ decreased in all three replicate pairs (Δ$R_{g}$ = −0.046 ± 0.065 nm), whereas the median-based shift was not sign-consistent (−0.025, −0.163 and +0.003 nm; Table S2), because the compaction developed progressively and to a replicate-dependent extent—most strongly in replicate R-2, which reached a compact subpopulation at $R_{g}$ ≈ 3.8 nm (median 3.843 nm; Table S1). Glucose therefore does not eliminate the conformational heterogeneity of HK1 (H) but redistributes the ensemble toward more compact substates. In human HK2, the median $R_{g}$ was unchanged within the replicate scatter (3.964 ± 0.020 versus 3.981 ± 0.074 nm; Δ$R_{g}$ = +0.017 ± 0.074 nm, not sign-consistent) and its interquartile range increased only modestly (0.079 ± 0.051 versus 0.095 ± 0.003 nm), so that the glucose response of this isoform is expressed in the shape of the local basin and in the backbone deviation rather than in its global size (see below). In the yeast enzymes the $R_{g}$ distributions were narrow and changed only slightly. In HK1 (Y), both distributions were bimodal under the peak-prominence criterion (Figure 3), and glucose lowered the replicate-averaged median $R_{g}$ from 2.402 ± 0.002 to 2.385 ± 0.015 nm (Δ$R_{g}$ = −0.017 ± 0.016 nm), most clearly in replicates R-1 and R-2 (−0.018 and −0.033 nm), whereas R-3 remained at the free-state value (−0.0004 nm; Table S2); it also roughly halved the interquartile range within each replicate (0.022 ± 0.002 versus 0.012 ± 0.002 nm, with non-overlapping replicate ranges; Table S1). HK2 (Y) shifted marginally toward larger values (2.433 ± 0.026 versus 2.454 ± 0.008 nm; Δ$R_{g}$ = +0.021 ± 0.031 nm, not sign-consistent). For HK2 (Y), the glucose-bound simulations started from the closed model built on the PI template (Section 2.1); over the three replicates its $R_{g}$ remained at, or marginally above, the value of the free/open enzyme, so that the closure of the inter-domain cleft modeled here was not accompanied by a measurable global contraction on the 100 ns timescale, and the small Δ$R_{g}$ of HK2 (Y) reflects the global size of a closed-cleft complex rather than an open→closed transition, which was not sampled. Overall, glucose redistributes the $R_{g}$ ensemble of the human enzymes—most clearly through the progressive compaction of HK1 (H)—whereas the $R_{g}$ distributions of the yeast enzymes change only slightly—a small compaction of HK1 (Y) and a marginal expansion of HK2 (Y).

**Figure 4:**
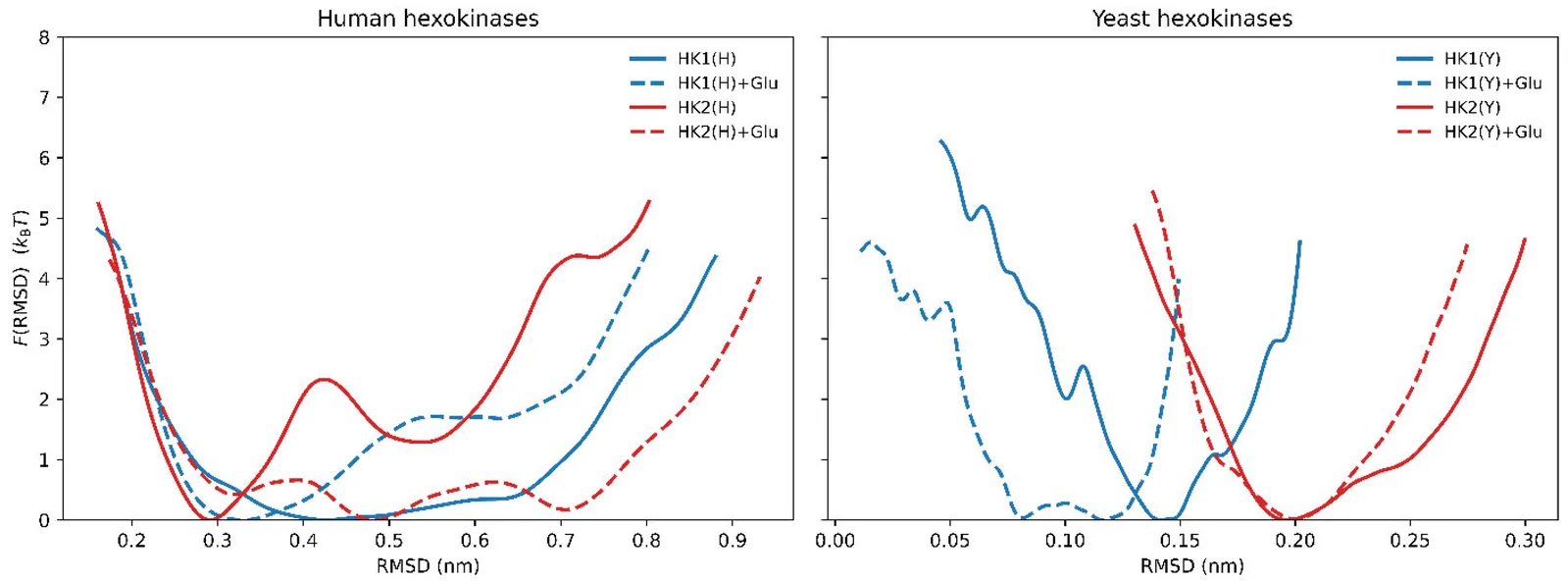
One-dimensional potential of mean force along the backbone RMSD, *F*(RMSD), obtained by Boltzmann inversion of the kernel-density estimate of the frames of the three replicates pooled for each condition, for the human (left) and yeast (right) hexokinases. Within each panel HK1 is shown in blue and HK2 in red; free states are drawn as solid lines and glucose-bound states (+Glu) as dashed lines. Energies are given in units of *k*_B_T relative to the global minimum of each profile, and grid points whose kernel density falls below 0.1% of its maximum are omitted. Approximate positions of the principal minima: HK1 (H), broad minimum at ≈0.40–0.55 nm (free) and ≈0.32 nm (+Glu); HK2 (H), ≈0.29 nm with a secondary well at ≈0.55 nm behind a ≈2.3 *k*_B_*T* barrier (free) and three shallow wells at ≈0.33, ≈0.48 and ≈0.70 nm within ≈0.6 *k*_B_T (+Glu); HK1 (Y), ≈0.14–0.15 nm (free) and a broad minimum at ≈0.08–0.12 nm (+Glu); HK2 (Y), ≈0.20 nm in both states.

The one-dimensional free-energy profile along $R_{g}$ follows by Boltzmann inversion of the $P(R_{g})$ distributions in Figure 3, whereas the profile along the backbone RMSD, *F*(RMSD), is shown explicitly in Figure 4 for the pooled frames of the three replicates. In human HK1, the free-state profile presents a broad, flat minimum spanning RMSD ≈ 0.40–0.55 nm; glucose shifts the minimum to ≈0.32 nm and leaves only a shallow shoulder near 0.55–0.62 nm (≈1.7 *k*_B_T), consistent with the reduction in the median RMSD in all three replicate pairs. In human HK2, the free-state profile shows a deep minimum at ≈0.29 nm separated by a barrier of ≈2.3 *k*_B_T (at ≈0.42 nm) from a secondary well at ≈0.55 nm (≈1.3 *k*_B_T); in the presence of glucose the profile flattens into three shallow wells at ≈0.33, ≈0.48 and ≈0.70 nm lying within ≈0.6 *k*_B_T of one another and separated by barriers below ≈0.7 *k*_B_T. Glucose therefore lowers the barriers between the RMSD substates of HK2 (H) and distributes the enzyme over a wider range of structural deviations, in agreement with the 2.8-fold broadening of its RMSD distribution, rather than homogenizing the ensemble. In the yeast enzymes, HK2 (Y) presents a single narrow well at ≈0.20 nm that is nearly superimposable in both conditions, whereas in HK1 (Y) glucose displaces the profile toward lower RMSD: the free-state minimum at ≈0.14–0.15 nm (with a shallow secondary well at ≈0.10 nm, ≈2 *k*_B_T) is replaced by a broad, flat minimum spanning ≈ 0.08–0.12 nm, formed by two wells within ≈0.3 *k*_B_T of each other.

**Figure 5:**
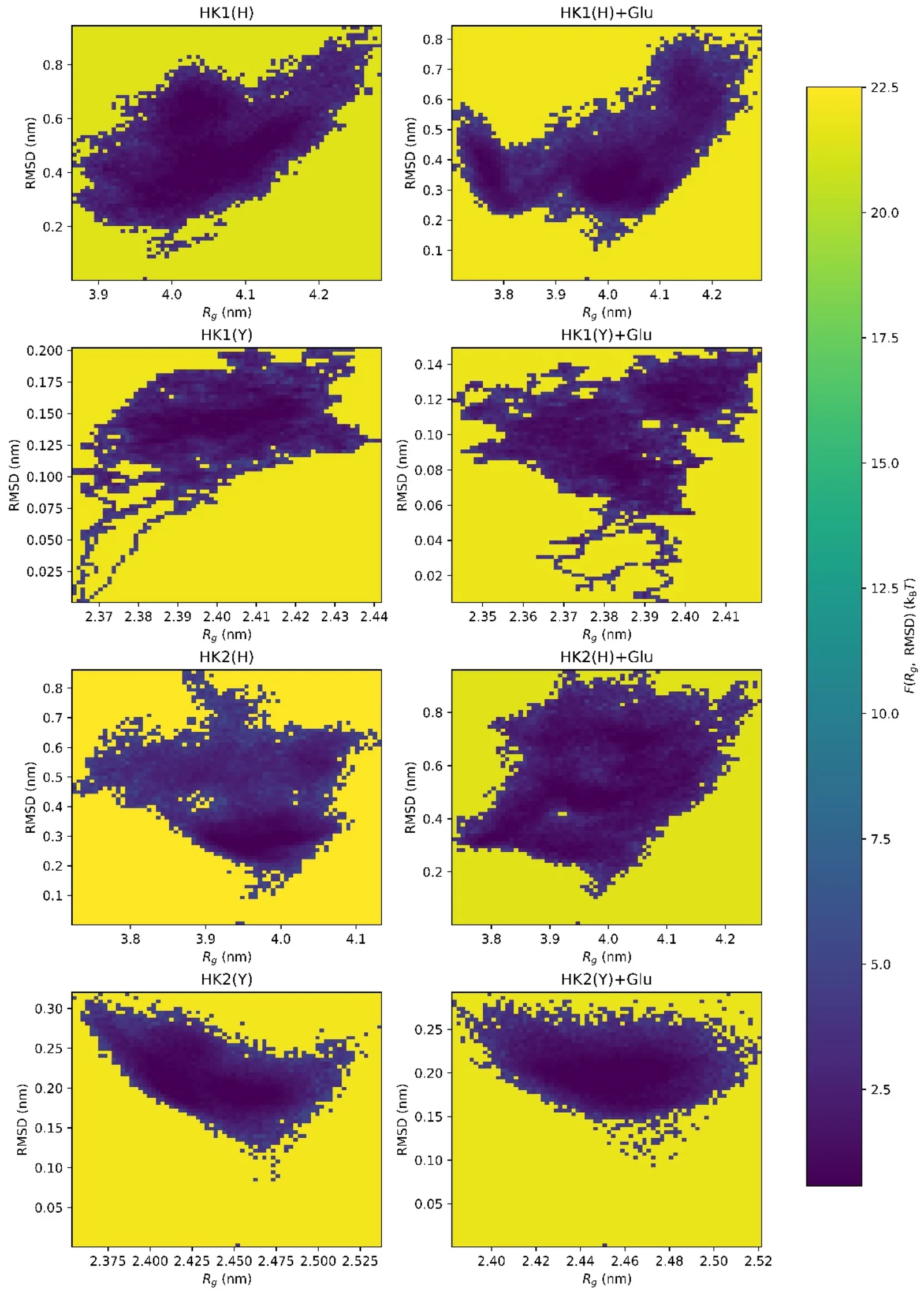
Two-dimensional potentials of mean force, *F*($R_{g}$, RMSD), computed from 70 × 70 two-dimensional histograms of the frame-matched ($R_{g}$, RMSD) pairs pooled over the three replicates, for HK1 (H), HK1 (Y), HK2 (H) and HK2 (Y) (top to bottom) in the free (left) and glucose-bound (+Glu, right) states. Free energy (colour bar, in units of *k*_B_T) is referred to the global minimum of each panel; darker regions correspond to lower free energy (higher population), and the uniform yellow background corresponds to unsampled bins.

The two-dimensional PMF *F*($R_{g}$, RMSD) provides a more complete view of the coupling between global compaction and structural deviation (Figure 5). In human HK1, the free-state landscape presents a broad, diagonally elongated basin connecting compact, low-RMSD states ($R_{g}$ ≈ 4.0 nm, RMSD ≈ 0.3–0.4 nm) with extended, higher-RMSD states ($R_{g}$ ≈ 4.15–4.2 nm, RMSD ≈ 0.5–0.6 nm). In the presence of glucose, this landscape shows a principal basin at $R_{g}$ ≈ 3.95–4.05 nm and RMSD ≈ 0.3 nm, a separate compact region at $R_{g}$ ≈ 3.75–3.85 nm (RMSD ≈ 0.3–0.45 nm), sampled mainly by replicate R-2, and a residual extended region near $R_{g}$ ≈ 4.15 nm (RMSD ≈ 0.65 nm), consistent with the progressive and replicate-dependent compaction described above. In human HK2, the free-state landscape is dominated by a basin at $R_{g}$ ≈ 3.95–4.0 nm and RMSD ≈ 0.3 nm with a secondary, higher-RMSD region (≈0.5–0.6 nm); in the presence of glucose the populations spread over a broader region ($R_{g}$ ≈ 3.8–4.2 nm, RMSD ≈ 0.3–0.8 nm) with the lowest free energies at RMSD ≈ 0.5–0.7 nm, reflecting a broadening of the basin rather than its homogenization. In HK1 (Y), the free-state landscape is a single basin at $R_{g}$ ≈ 2.38–2.43 nm and RMSD ≈ 0.12–0.16 nm, whereas in the presence of glucose it is displaced toward smaller $R_{g}$ and lower RMSD ($R_{g}$ ≈ 2.37–2.40 nm, RMSD ≈ 0.07–0.10 nm), with a secondary region at $R_{g}$ ≈ 2.39–2.41 nm and RMSD ≈ 0.11–0.13 nm sampled mainly by replicate R-3 (Figure S1); the thin tails at very low RMSD correspond to the first frames of the trajectories. In HK2 (Y), the landscape remains a single, compact basin in both conditions (RMSD ≈ 0.15–0.27 nm), shifted by glucose toward slightly larger $R_{g}$ (≈2.42–2.50 nm versus ≈2.39–2.48 nm), confirming that glucose binding does not substantially alter the topology of the global energy landscape in this system.

**Figure 6:**
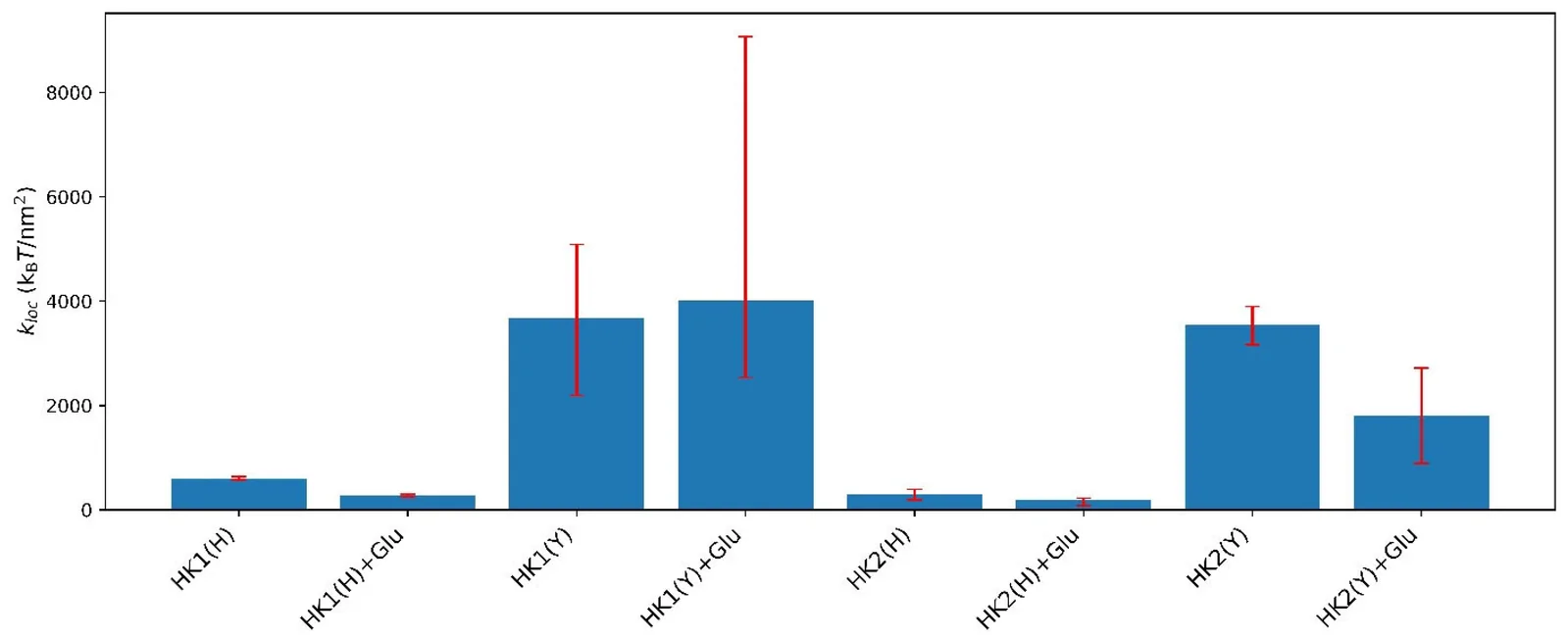
Local stiffness of the principal minimum of the $R_{g}$ free-energy profile ($k_{loc}$, in units of *k*_B_T/nm^2^) for each system and condition, estimated from the frames of the three replicates pooled for each condition. Bars give the bootstrap mean and red error bars the 95% bootstrap confidence interval (*n* = 400 resamples): HK1 (H), 605 [571–643] versus 279 [250–305]; HK1 (Y), 3674 [2198–5088] versus 4006 [2540–9066]; HK2 (H), 303 [189–394] versus 194 [81–226]; HK2 (Y), 3544 [3171–3901] versus 1803 [896–2723] *k*_B_T/nm^2^ (free versus +Glu). The between-replicate means ± SD and the individual replicate values are given in Table 1 and Table S3.

The local curvature $k_{loc}$ of the principal minimum of the $R_{g}$ PMF quantifies the width of the dominant well (Figure 6; Table 1). Because $k_{loc}$ scales with the inverse squared width of the $R_{g}$ distribution, its absolute value is size-dependent—those of the compact yeast monomers (≈10^3^–10^4^ *k*_B_T/nm^2^) exceed those of the two-domain human enzymes (≈10^2^–10^3^ *k*_B_T/nm^2^)—and the interspecies comparison is therefore made on the glucose/free ratio. The most robust effect was observed in HK2 (H), in which glucose reduced $k_{loc}$ from 980 ± 145 to 295 ± 112 *k*_B_T/nm^2^ (between-replicate mean ± SD; ratio 0.30) with non-overlapping replicate ranges (826–1115 versus 194–415 *k*_B_T/nm^2^), indicating a substantially wider, more permissive dominant well; the pooled estimate pointed in the same direction (303 versus 194 *k*_B_T/nm^2^; ratio 0.64). In HK1 (H), the curvature of the dominant well was not altered beyond the run-to-run dispersion (743 ± 543 versus 892 ± 208 *k*_B_T/nm^2^), whereas the pooled estimate decreased 2.2-fold (605 [571–643] versus 279 [250–305] *k*_B_T/nm^2^); this discrepancy arises because the glucose-bound replicates sampled the well at different degrees of compaction (Figure 1), which widens the pooled distribution without widening the well sampled within each run. In the yeast enzymes the two isoforms responded in opposite directions, each consistently across the three replicate pairs but with overlapping replicate ranges: HK1 (Y) stiffened (11,871 ± 7561 versus 27,341 ± 16,755 *k*_B_T/nm^2^; ratio 2.30; replicate ranges 7347–20,599 versus 11,644–44,985 *k*_B_T/nm^2^), although the pooled estimate, broadened by the offsets between replicates, barely changed (3674 versus 4006 *k*_B_T/nm^2^; ratio 1.09, overlapping CIs), and HK2 (Y) softened (4514 ± 1783 versus 3109 ± 1311 *k*_B_T/nm^2^; ratio 0.69; pooled ratio 0.51). Because the between-replicate coefficients of variation (15–73%) are of the same order as several of these glucose effects, only the softening of HK2 (H) is regarded as robust at *n* = 3, and the opposite, sign-consistent responses of the two yeast enzymes are regarded as indicative.

The explicit quantification of basin occupancy and apparent residence times (Table 2) adds a thermodynamic and effective kinetic dimension to this picture. Because the pooled free-state $R_{g}$ PMF of HK1 (H), HK2 (H) and HK2 (Y) was unimodal under the peak-prominence criterion (Section 2.5), their dominant basin corresponds to the region within 2 *k*_B_T of the minimum, which contained 94–96% of the free-state frames on average. In HK1 (Y) the free-state PMF was bimodal (Figure 3), and the compact-side limit of the basin is the free-energy maximum that separates the dominant minimum from a secondary, more compact one; because this maximum lies only 0.23 *k*_B_T above the minimum—well below the thermal energy—the HK1 (Y) basin (2.396–2.432 nm) contained only 63% of the free-state frames (0.630 ± 0.035), and its occupancy measures the population on the expanded side of a shallow maximum rather than residence in a kinetically separated state. In human HK2, glucose lowered the occupancy of this basin in all three replicate pairs (0.942 ± 0.096 versus 0.738 ± 0.040, with non-overlapping replicate ranges of 0.831–0.998 and 0.712–0.784), with excursions on both sides of the basin, and shortened the apparent residence time from 25.7 ± 24.0 to 1.6 ± 0.5 ns, again in all three pairs; glucose therefore makes the HK2 (H) basin markedly more labile, consistent with its softer well and broadened RMSD distribution. In human HK1, the mean loss of occupancy (−0.202 ± 0.342) was driven by replicate R-2 (occupancy 0.351; ART 1.4 ns), whose compact subpopulation at $R_{g}$ ≈ 3.8 nm lies outside the basin, whereas in the other two replicate pairs the occupancy changed by at most 0.01, and the ART did not change beyond the run-to-run dispersion (9.1 ± 9.4 versus 9.5 ± 13.4 ns). In HK2 (Y) the occupancy was unchanged (0.958 ± 0.034 versus 0.955 ± 0.008). In HK1 (Y), glucose lowered the occupancy in replicates R-1 and R-2 (0.090 and 0.003) but not in R-3 (0.695 versus 0.607 in the free state; mean 0.630 ± 0.035 versus 0.263 ± 0.377), and shortened the ART in all three pairs, with non-overlapping replicate ranges (2.9 ± 0.9 versus 0.6 ± 0.6 ns; 2.1–3.9 versus 0.15–1.3 ns). Given the shallow compact-side limit of this basin, these changes reflect the displacement of the glucose-bound replicates toward the adjacent, more compact minimum (Figure 3) rather than escape over an appreciable barrier; replicate R-2 visited the basin in only two short segments, so its ART is not informative. The ART of HK2 (Y) was shorter in the glucose-bound state in all three pairs (8.4 ± 7.1 versus 3.8 ± 0.1 ns), but by less than 0.6 ns in two of them and without loss of occupancy; this reflects more frequent crossings of the upper basin edge as the glucose-bound ensemble shifts toward larger $R_{g}$ (Δ$R_{g}$ = +0.021 ± 0.031 nm) rather than a depletion of the basin. Long ART values remain bounded by the 100 ns trajectory length in replicates with few exits (Table S5).

The separation metrics between the free and glucose-bound distributions (Table 3) were evaluated against the variability between independent runs. Between the pooled ensembles, the JSD of $R_{g}$ was 0.115 for both human enzymes, 0.139 for HK2 (Y) and 0.222 for HK1 (Y), and the JSD of the RMSD was largest for HK1 (Y) (0.504), followed by HK2 (H) (0.237), HK1 (H) (0.097) and HK2 (Y) (0.038). However, except in HK1 (Y), the distances between individual free and +Glu replicates were of the same order as those between replicates of the same condition: the ratio of cross-pair to between-replicate JSD ranged from 0.81 to 1.18 for $R_{g}$ and from 0.89 to 1.28 for the RMSD, with the highest value for the RMSD of HK2 (H), and the BD gave the same picture (ratios 0.82–1.18). HK1 (Y) was the only system in which the cross-pair distances exceeded this baseline (JSD ratios 1.39 for $R_{g}$ and 1.97 for the RMSD; BD ratios 1.44 and 2.14), in agreement with the lower RMSD and slightly smaller $R_{g}$ of its glucose-bound ensemble (Figure 3, Figure 4 and Figure 5), although the baseline itself is broad (RMSD JSD 0.324 ± 0.340). The pooled distances are smaller than the cross-pair distances because pooling averages out replicate-specific offsets. Hence, on the 100 ns timescale, the whole-distribution differences between the free and glucose-bound ensembles do not exceed the run-to-run variability in any system other than HK1 (Y), and these metrics do not by themselves support a ranking of the ligand impact; among the other systems, the RMSD of HK2 (H), which shows the largest ratio, is consistent with the glucose effects found for this isoform with $k_{loc}$ (robust), $\kappa_{T}^{eff}$ (indicative) and basin occupancy (robust).

The effective volumetric compliance $\kappa_{T}^{eff}$ (Table 1) complements these descriptors. Because it is derived from the variance of the cavity volume computed from $R_{g}$, its absolute value is size-dependent—values for the human enzymes (≈2–12 × 10^−2^ bar^−1^) exceed those of the yeast monomers (≈0.4–4 × 10^−3^ bar^−1^) by one to more than two orders of magnitude—and only within-system glucose/free ratios are compared between species. In both human isoforms glucose increased $\kappa_{T}^{eff}$: in HK1 (H) from (24.1 ± 15.5) × 10^−3^ to (66.9 ± 59.6) × 10^−3^ bar^−1^ (between-replicate mean ± SD; ratio 2.8), with a pooled estimate rising from 38.9 to 123.8 × 10^−3^ bar^−1^ (ratio 3.2; non-overlapping 95% CIs), and in HK2 (H) from (25.3 ± 25.2) × 10^−3^ to (44.6 ± 4.8) × 10^−3^ bar^−1^ (ratio 1.8), with a pooled estimate rising from 27.2 to 68.4 × 10^−3^ bar^−1^ (ratio 2.5; non-overlapping 95% CIs). The between-replicate dispersion was large in free HK2 (H) and in glucose-bound HK1 (H)—in each case dominated by replicate R-2 (54.3 and 134.7 × 10^−3^ bar^−1^, respectively)—so that the replicate ranges overlap; the direction of the effect was nevertheless the same for both estimators and both human isoforms. In HK1 (H), the larger compliance accompanies the progressive compaction and broadening of the $R_{g}$ distribution described above while the curvature of the dominant well is preserved; that is, glucose enlarges the global volumetric fluctuations of HK1 (H) by populating additional, more compact states rather than by softening the well itself. In HK2 (H), the larger compliance is consistent with the threefold reduction in $k_{loc}$ (Table 1) and with the broadened RMSD distribution and lowered inter-well barriers (Figure 1 and Figure 4), indicating that glucose renders the HK2 (H) basin volumetrically more permissive rather than rigidifying it.

In the yeast enzymes the glucose response of $\kappa_{T}^{eff}$ was opposite to the human response in HK1 (Y) and estimator-dependent in HK2 (Y) (Table 1). In HK1 (Y), glucose reduced the compliance in all three replicates, with non-overlapping replicate ranges [(0.919 ± 0.061) × 10^−3^ versus (0.365 ± 0.101) × 10^−3^ bar^−1^; ratio 0.40; 0.851–0.971 versus 0.273–0.472 × 10^−3^ bar^−1^], whereas the pooled estimate did not change [0.943 (0.871–1.002) versus 1.008 (0.931–1.080) × 10^−3^ bar^−1^; ratio 1.07]. The pooled value is inflated by the offset between the glucose-bound replicates, which fluctuate around different positions (median $R_{g}$ 2.371–2.400 nm, versus 2.400–2.404 nm in the free state; Table S1), so the within-replicate estimate is the informative one here: glucose narrows the volumetric fluctuations of HK1 (Y) within each run, consistent with the halved interquartile range of its $R_{g}$ and the stiffening of its well, while displacing the replicates to slightly different positions within the $R_{g}$ distribution. In HK2 (Y), the between-replicate mean increased [(1.49 ± 0.12) × 10^−3^ versus (2.07 ± 0.47) × 10^−3^ bar^−1^; ratio 1.39] whereas the pooled estimate decreased (3.66 versus 2.20 × 10^−3^ bar^−1^; ratio 0.60); the pooled free-state value is inflated by the offset between the median $R_{g}$ of the three free-state replicates (2.411–2.462 nm), so that no robust glucose effect can be assigned to HK2 (Y). Glucose therefore increases the global volumetric fluctuations of both human enzymes ($\kappa_{T}^{eff}$ ratios of 1.8–3.2 for both estimators) but reduces those of HK1 (Y) within each replicate (ratio 0.40) and changes those of HK2 (Y) inconsistently (ratios 0.60–1.39, depending on the estimator).

The interpretation of our results in terms of basin reweighting and changes in basin shape is consistent with the general framework of population shift, in which ligand binding modifies the relative populations of conformational substates and reconfigures the connectivity of the landscape [9]. Classic studies of enzymes with functional transitions, such as adenylate kinase and dihydrofolate reductase, show that ligands can favor specific global states and alter free-energy profiles without necessarily implying uniform local rigidization, but rather changes in the width and accessibility of the basins [38,39,40]. From the methodological point of view, the characterization of metastable states and their relative kinetic stability via landscapes and transition networks is widely supported in state-based and molecular kinetics analyses [41]. Finally, the use of volumetric descriptors derived from fluctuations as indicators for structural flexibility finds support in studies of protein compressibility based on MD simulations [42,43].

## 4. Conclusions

Taken together, the integrated analysis of KDE distributions of $R_{g}$, one-dimensional PMFs, the two-dimensional free-energy landscape $F\left(R_{g},\;RMSD\right)$, local stiffness $k_{loc}$, basin occupancy and residence time metrics, and effective volumetric compliance $\kappa_{T}^{eff}$ shows that glucose binding differentially modulates the local conformational flexibility and free-energy basin shape of human and yeast hexokinases. In human isoforms, glucose enlarges the global volumetric fluctuations of the local ensemble ($\kappa_{T}^{eff}$ 1.8–3.2-fold higher than in the free state): in HK1 it drives a progressive, replicate-dependent compaction toward more compact substates, consistent with a hinge-like closure of its two halves, while preserving the curvature of the dominant well, whereas in HK2 it softens the dominant well about threefold, lowers the occupancy of its free-state basin and shortens its residence time in all three replicates and broadens the distribution of structural deviations, lowering the barriers between RMSD substates. In contrast, yeast hexokinases respond weakly or in the opposite direction: in HK1 (Y) glucose slightly compacts the ensemble, keeps the backbone closer to its starting conformation and narrows the volumetric fluctuations within each replicate, while HK2 (Y) presents minimal changes in the topology of the energy landscape. Because several of these effects are comparable to the between-replicate dispersion (*n* = 3), only the softening and basin destabilization of HK2 and, in HK1 (Y), the lower backbone deviation and narrower within-replicate fluctuations meet the robustness criterion, whereas the larger volumetric fluctuations of the human enzymes are indicative; whole-distribution distances between the free and glucose-bound ensembles exceeded the between-replicate variability only in HK1 (Y), and longer or more numerous simulations will be required to quantify the remaining effects.

Future work should address the limits of the present sampling and models through enhanced-sampling simulations (metadynamics or replica exchange) using $R_{g}$ and the inter-lobe cleft distance as collective variables, to sample the open↔closed transition of HK2 (Y) and the non-stationary compaction of HK1 (H); larger pools of independent replicates (e.g., ten 100 ns runs per condition), to bring whole-distribution distances beyond the run-to-run variability; single-site (C-terminal-only) simulations of HK1, to partition its glucose response between the catalytic and non-catalytic sites; and ensembles in which unresolved segments are completed with AlphaFold or MODELLER.

These results indicate that, although the catalytic function of hexokinases is conserved, the coupling between glucose binding, modulation of local conformational flexibility, kinetic stability of dominant basins, and global volumetric flexibility was not conserved between isoforms or between species in the present simulations. These differences in the shape of the local free-energy basins are compatible with a divergence of the glucose-dependent conformational dynamics between the human and yeast enzymes. Given the relevance of human HK1 and HK2 as anticancer drug targets, these findings suggest that computational inhibitor design should rely on human-specific structural ensembles rather than yeast models, despite their high sequence homology.

## Figures and Tables

**Table 1:** Conformational and fluctuation-derived descriptors of each system and condition from three independent 100 ns replicates. $R_{g}$ and RMSD: median of each replicate, given as the mean ± SD across replicates. $k_{loc}$ and $\kappa_{T}^{eff}$: between-replicate mean ± SD of the per-replicate estimates (*n* = 3), and estimate from the frames of the three replicates pooled for each condition with its 95% bootstrap confidence interval (CI; $k_{loc}$: 400 resamples; $\kappa_{T}^{eff}$: moving-block bootstrap, 1000 resamples). Per-replicate values are given in Tables S1 and S3. H: human isoform; Y: yeast isoform; free: ligand-free condition; +Glu: glucose-bound condition.

System	$R_{g}$ (nm)	RMSD (nm)	$k_{loc}$_c_ (*k*_B_T/nm^2^) Mean ± SD	$k_{loc}$ (*k*_B_T/nm^2^) Pooled [95% CI]	$\kappa_{T}^{eff}$ (10^−3^ bar^−1^) Mean ± SD	$\kappa_{T}^{eff}$ (10^−3^ bar^−1^) Pooled [95% CI]
HK1 (H)	4.052 ± 0.058	0.496 ± 0.101	743 ± 543	605 [571–643]	24.1 ± 15.5	38.9 [35.7–42.1]
HK1 (H) +Glu	3.990 ± 0.139	0.387 ± 0.072	892 ± 208	279 [250–305]	66.9 ± 59.6	123.8 [114.9–133.8]
HK1 (Y)	2.402 ± 0.002	0.143 ± 0.004	11,871 ± 7561	3674 [2198–5088]	0.919 ± 0.061	0.943 [0.871–1.002]
HK1 (Y) +Glu	2.385 ± 0.015	0.102 ± 0.022	27,341 ± 16,755	4006 [2540–9066]	0.365 ± 0.101	1.008 [0.931–1.080]
HK2 (H)	3.964 ± 0.020	0.376 ± 0.132	980 ± 145	303 [189–394]	25.3 ± 25.2	27.2 [24.6–29.9]
HK2 (H) +Glu	3.981 ± 0.074	0.534 ± 0.131	295 ± 112	194 [81–226]	44.6 ± 4.8	68.4 [63.7–72.9]
HK2 (Y)	2.433 ± 0.026	0.211 ± 0.017	4514 ± 1783	3544 [3171–3901]	1.49 ± 0.12	3.66 [3.39–3.92]
HK2 (Y) +Glu	2.454 ± 0.008	0.201 ± 0.008	3109 ± 1311	1803 [896–2723]	2.07 ± 0.47	2.20 [2.05–2.33]

**Table 2:** Occupancy of the dominant $R_{g}$ basin and apparent residence time (ART) in the free and glucose-bound states, from three independent 100 ns replicates. The dominant basin is the region within 2 *k*_B_T of the minimum of the pooled free-state $R_{g}$ PMF (Section 2.5), applied to both conditions; occupancy and ART are given as the between-replicate mean ± SD, and Δ values as the mean ± SD of the three replicate-pair differences. ΔOccupancy had the same sign in all three pairs only for HK2 (H), and ΔART for HK1 (Y), HK2 (H) and HK2 (Y). In HK1 (Y), the compact-side limit of the basin is a shallow free-energy maximum (0.23 *k*_B_T above the minimum). Per-replicate values are given in Table S5. H: human isoform; Y: yeast (*Saccharomyces cerevisiae*) isoform.

System	Dominant Basin ($R_{g}$, nm)	Occupancy Free	Occupancy + Glu	ΔOccupancy	ART Free (ns)	ART + Glu (ns)	ΔART (ns)
HK1 (H)	[3.918, 4.187]	0.942 ± 0.055	0.739 ± 0.342	−0.202 ± 0.342	9.1 ± 9.4	9.5 ± 13.4	+0.4 ± 4.2
HK1 (Y)	[2.396, 2.432]	0.630 ± 0.035	0.263 ± 0.377	−0.368 ± 0.402	2.9 ± 0.9	0.6 ± 0.6	−2.2 ± 1.1
HK2 (H)	[3.846, 4.072]	0.942 ± 0.096	0.738 ± 0.040	−0.203 ± 0.086	25.7 ± 24.0	1.6 ± 0.5	−24.0 ± 23.7
HK2 (Y)	[2.383, 2.489]	0.958 ± 0.034	0.955 ± 0.008	−0.003 ± 0.041	8.4 ± 7.1	3.8 ± 0.1	−4.6 ± 7.1

**Table 3:** Jensen–Shannon divergence (JSD, base 2) and Bhattacharyya distance (BD) between the free and +Glu distributions of $R_{g}$ and RMSD. Pooled: distance between the ensembles pooled over the three replicates. Cross: mean ± SD over the nine pairs of individual free and +Glu replicates. Baseline: mean ± SD over the six pairs of replicates of the same condition. Ratio: cross/baseline; values close to 1 indicate that the free/+Glu difference does not exceed the between-replicate variability. Distances were computed from kernel-density estimates on a common grid (Section 2.5); histogram-based values and the BD cross and baseline values are given in Table S6. H: human isoform; Y: yeast isoform.

Coordinate	System	JSD Pooled	BD Pooled	JSD Cross (Mean ± SD)	JSD Baseline (Mean ± SD)	JSD Ratio	BD Ratio
$R_{g}$	HK1 (H)	0.115	0.112	0.305 ± 0.190	0.377 ± 0.130	0.81	0.82
	HK1 (Y)	0.222	0.205	0.382 ± 0.235	0.274 ± 0.289	1.39	1.44
	HK2 (H)	0.115	0.097	0.251 ± 0.110	0.227 ± 0.136	1.10	1.18
	HK2 (Y)	0.139	0.111	0.296 ± 0.229	0.252 ± 0.293	1.18	1.02
RMSD	HK1 (H)	0.097	0.071	0.240 ± 0.198	0.219 ± 0.138	1.10	1.15
	HK1 (Y)	0.504	0.552	0.636 ± 0.234	0.324 ± 0.340	1.97	2.14
	HK2 (H)	0.237	0.196	0.492 ± 0.272	0.386 ± 0.349	1.28	1.17
	HK2 (Y)	0.038	0.028	0.099 ± 0.093	0.111 ± 0.086	0.89	0.91

## Data Availability

The molecular dynamics trajectories, analysis scripts, and derived data supporting the findings of this study are available from the corresponding author upon reasonable request.

## Supplementary Materials

The following supporting information can be downloaded at: https://www.mdpi.com/article/10.3390/biology15201900/s1. Figure S1: Time evolution of the backbone RMSD, radius of gyration $R_{g}$ and per-residue RMSF for the eight system–condition combinations; Figure S2: Mutual superposition of the three per-replicate representative (most-populated-cluster) structures for each system; Figure S3: Per-residue glucose-contact occupancy for each complex; Table S1: Per-replicate descriptors of the radius of gyration and of the backbone RMSD for each system and condition over the 100 ns production run; Table S2: Reproducibility of the glucose-induced change across replicate pairs; Table S3: Per-replicate local free-energy well stiffness $k_{loc}$ and effective volumetric compliance $\kappa_{T}^{eff}$; Table S4: Quantitative metrics of glucose persistence in each binding site over the 100 ns trajectory; Table S5: Per-replicate occupancy of the dominant $R_{g}$ basin and apparent residence time (ART); Table S6: Jensen–Shannon divergence (JSD) and Bhattacharyya distance (BD) between the free and +Glu distributions of $R_{g}$ and RMSD.
