# Supplementary material for: Glucose Modulation of the Local Conformational Flexibility and Free-Energy Basin Shape of Human and Yeast Hexokinases: A Comparative Molecular Dynamics Analysis
Source: Biology (Basel). 2026 Oct 21;15(20):1900. doi: 10.3390/biology15201900 (PMC13701726; doi:10.3390/biology15201900)
Supplement: Supplementary file 1 [file biology-15-01900-s001.zip › biology-4489762-supplementary.pdf]

Supplementary Material

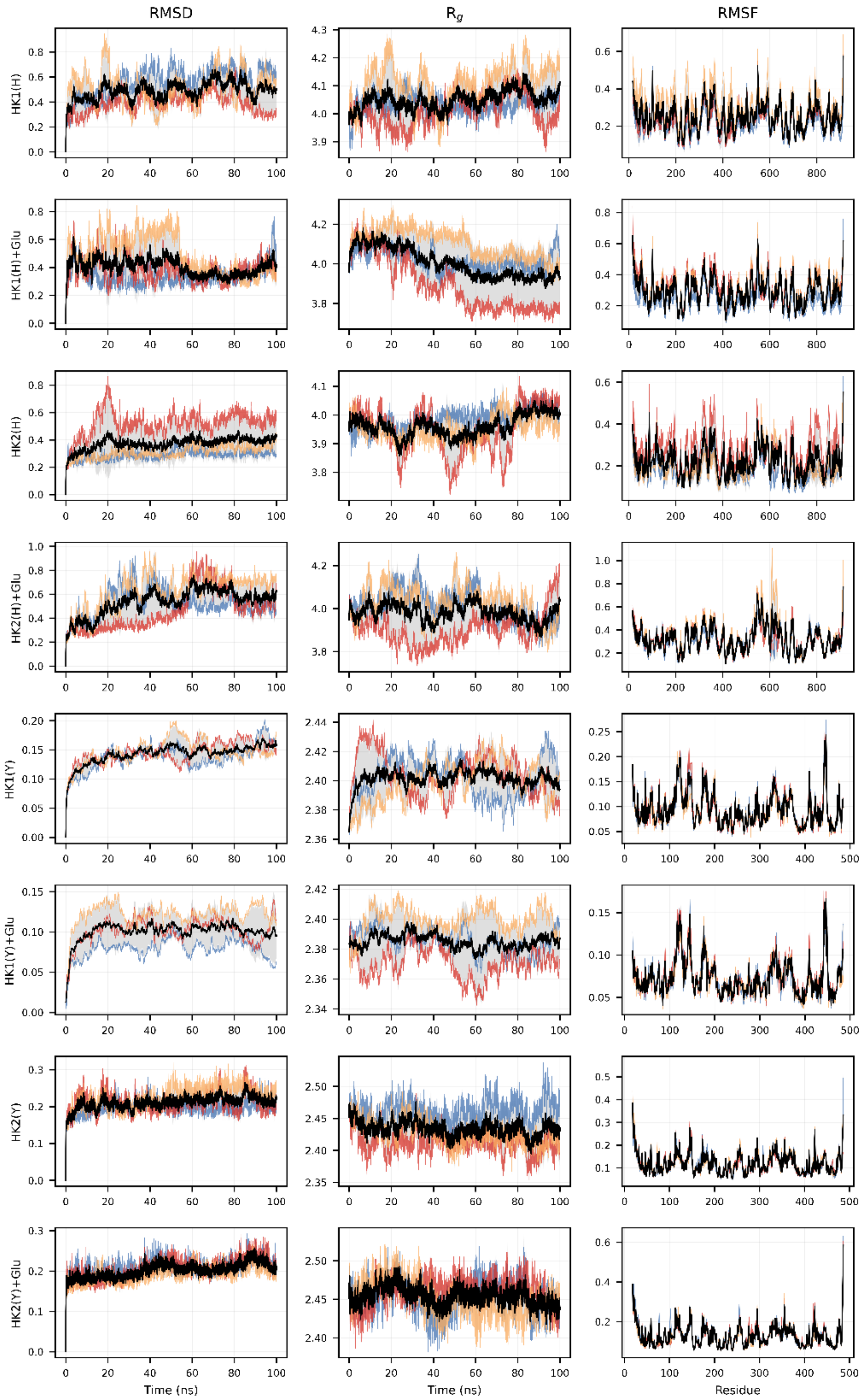

Figure S1. Time evolution of the backbone RMSD (left column), radius of gyration Rg (centre) and per-residue RMSF (right) for the eight system–condition combinations (rows: HK1 (H), HK2 (H), HK1 (Y), HK2 (Y), each in the free and +Glu states), over the 100 ns production trajectories. The three independent replicates are shown as thin coloured lines (blue, red, orange), with the replicate mean in black and the ±1 SD envelope shaded in grey. RMSD and Rg are plotted against simulation time (ns) and RMSF against residue number; the RMSF axis uses simulation-topology residue numbering (Table S4).

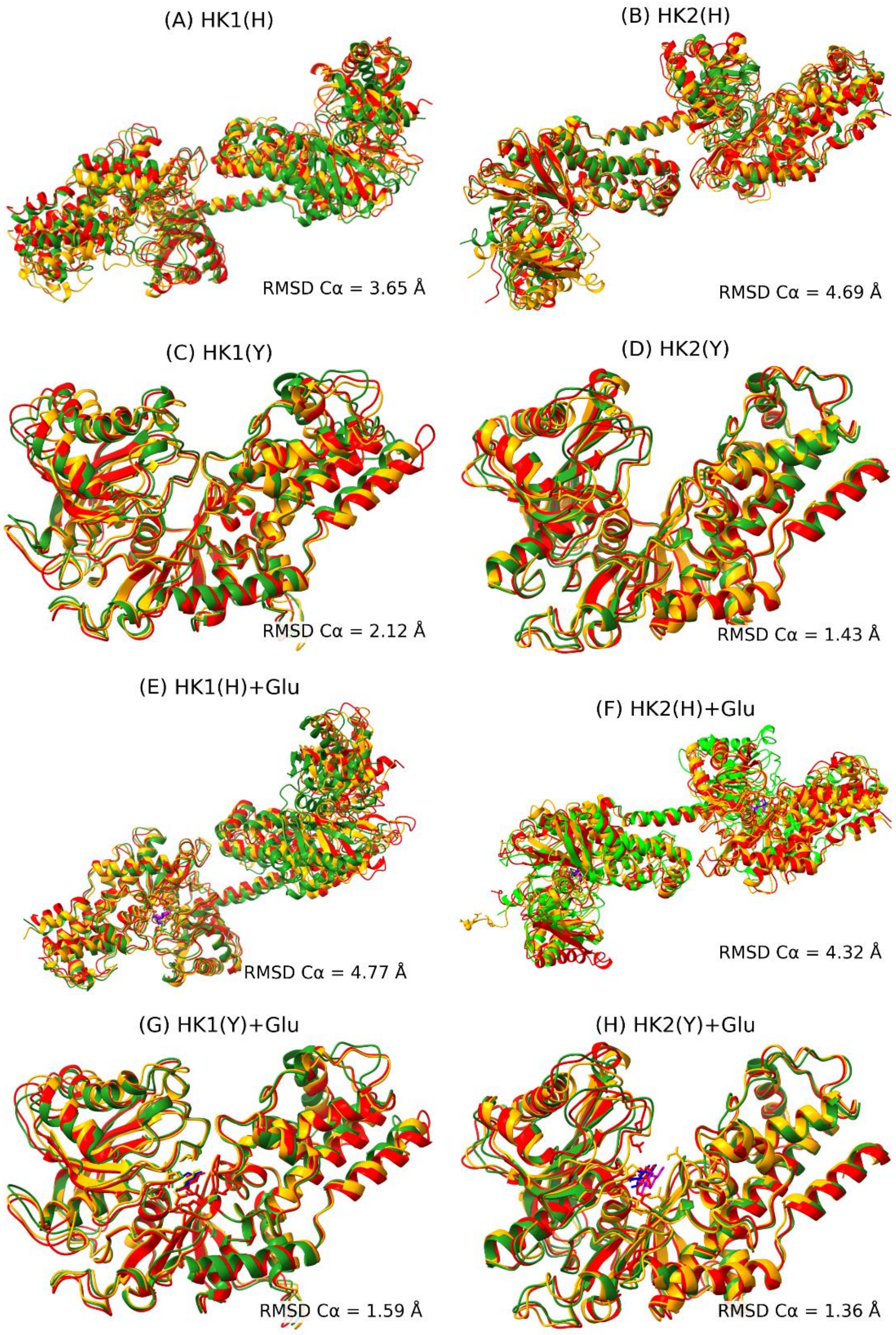

Figure S2. Mutual superposition of the three per-replicate representative (most-populated-cluster) structures for each system, used to assess inter-replicate convergence; the three replicates are coloured green, red and orange and glucose is shown as sticks. Panels (free): (A) HK1 (H), (B) HK2 (H), (C) HK1 (Y), (D) HK2 (Y); panels (+Glu): (E) HK1 (H), (F) HK2 (H), (G) HK1 (Y), (H) HK2 (Y). The pairwise-averaged inter-replicate Cα RMSD (Å) is given in each panel. The human enzymes converged in both conditions (free: 3.65 Å HK1 (H), 4.69 Å HK2 (H);

+Glu: 4.77 Å HK1 (H), 4.32 Å HK2 (H)), as did the yeast enzymes (free: 2.12 Å HK1 (Y), 1.43 Å HK2 (Y); +Glu: 1.59 Å HK1 (Y), 1.36 Å HK2 (Y)).

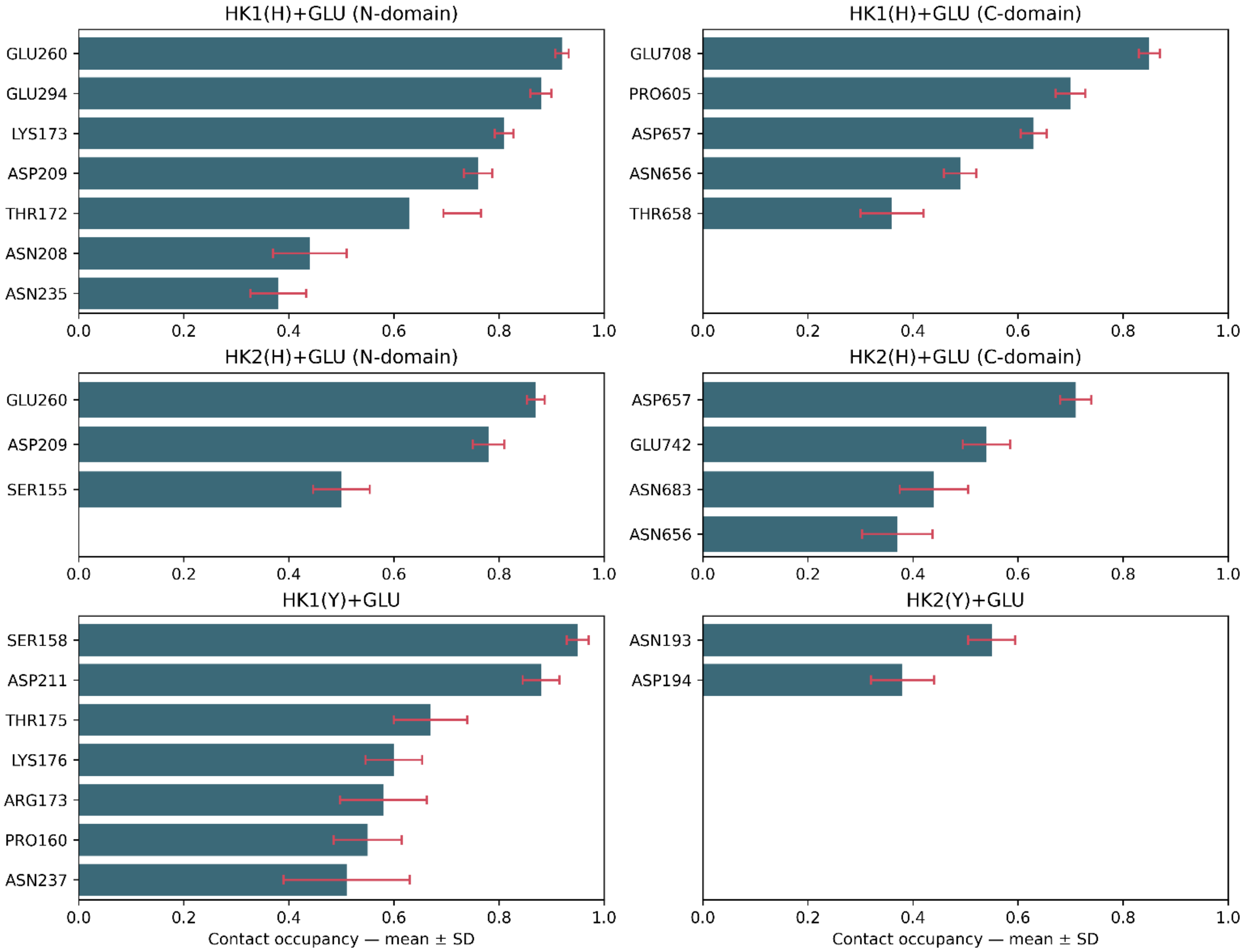

Figure S3. Per-residue glucose-contact occupancy for each complex, defined as the fraction of trajectory frames in which each pocket residue remained in contact with the ligand, averaged over the three replicates (bars: mean; error bars: ±1 SD between replicates); residues follow simulation-topology numbering (Table S4). The consensus (high-occupancy, low-variance) contacts were, in the human enzymes, the charged N-pocket cluster (Glu260, Glu294, Lys173, Asp209, Thr172 in HK1 (H); Glu260, Asp209, Ser155 in HK2 (H)) and the catalytic C-pocket anchors (Glu708, Pro605, Asp657 in HK1 (H); Asp657, Glu742 in HK2 (H)); in the single-domain yeast enzymes they were Asp211, Ser158, Thr175 and Lys176 in HK1 (Y) and Asn193 and Asp194 in HK2 (Y).

Table S1. Per-replicate descriptors of the radius of gyration and of the backbone RMSD for each system and condition over the 100 ns production run: median, interquartile range (IQR) and linear drift (least-squares slope versus time). R-1–R-3: independent replicates. These values underlie the between-replicate means ± SD reported for R_g and RMSD in Table 1 of the main text.

**System**	**Replicate**	**R_g median (nm)**	**R_g IQR (nm)**	**R_g slope (10^{-3} nm ns^{-1})**	**RMSD median (nm)**	**RMSD IQR (nm)**	**RMSD slope (10^{-3} nm ns^{-1})**
HK1 (H)	R-1	4.032	0.038	+0.14	0.588	0.159	+2.54
	R-2	4.006	0.079	+0.71	0.387	0.108	+0.57
	R-3	4.117	0.086	+0.81	0.512	0.164	+0.34
HK1 (H) +Glu	R-1	4.007	0.083	−0.81	0.322	0.076	+0.65
	R-2	3.843	0.221	−4.04	0.374	0.108	−0.41
	R-3	4.120	0.123	−2.05	0.464	0.244	−2.77
HK1 (Y)	R-1	2.404	0.024	−0.04	0.138	0.024	+0.47
	R-2	2.403	0.022	−0.15	0.145	0.024	+0.44
	R-3	2.400	0.020	+0.24	0.144	0.021	+0.31
HK1 (Y) +Glu	R-1	2.386	0.011	−0.13	0.080	0.013	+0.11
	R-2	2.371	0.014	−0.04	0.104	0.017	+0.13
	R-3	2.400	0.011	+0.03	0.123	0.014	+0.12
HK2 (H)	R-1	3.987	0.047	+0.87	0.289	0.038	+0.58
	R-2	3.956	0.138	+0.39	0.528	0.093	+1.14
	R-3	3.948	0.053	+0.43	0.312	0.062	+1.09
HK2 (H) +Glu	R-1	4.010	0.095	−1.30	0.507	0.122	+0.36
	R-2	3.896	0.091	+0.95	0.418	0.239	+4.00
	R-3	4.035	0.098	−0.91	0.677	0.180	+3.56
HK2 (Y)	R-1	2.462	0.024	+0.10	0.195	0.021	+0.19
	R-2	2.411	0.019	−0.15	0.207	0.032	+0.13
	R-3	2.426	0.025	−0.37	0.230	0.043	+0.71
HK2 (Y) +Glu	R-1	2.452	0.029	−0.05	0.207	0.026	+0.32
	R-2	2.463	0.023	+0.09	0.204	0.034	+0.59
	R-3	2.447	0.033	−0.45	0.192	0.027	+0.44

Slopes are given in units of 10^{-3} nm ns^{-1}; positive values indicate an increase over the trajectory. All trajectories were analysed at their native sampling of 0.01 ns (10,001 frames per trajectory).

Table S2. Reproducibility of the glucose-induced change across replicate pairs. For each nominal replicate pair, $\Delta X = X(\text{+Glu}) - X(\text{free})$ was computed for the median R_g, the time-averaged R_g and the median backbone RMSD; the last columns give the mean ± SD of the three differences and whether the three differences share the same sign.

**Descriptor**	**System**	**Δ R-1 (nm)**	**Δ R-2 (nm)**	**Δ R-3 (nm)**	**Mean ± SD (nm)**	**Sign-consistent**
Median R_g	HK1 (H)	−0.025	−0.163	+0.003	−0.062 ± 0.089	No
	HK1 (Y)	−0.018	−0.033	−0.0004	−0.017 ± 0.016	Yes
	HK2 (H)	+0.023	−0.060	+0.087	+0.017 ± 0.074	No
	HK2 (Y)	−0.010	+0.052	+0.021	+0.021 ± 0.031	No
Time-averaged R_g	HK1 (H)	−0.013	−0.122	−0.004	−0.046 ± 0.065	Yes
	HK1 (Y)	−0.017	−0.034	+0.001	−0.017 ± 0.017	No
	HK2 (H)	+0.030	−0.046	+0.074	+0.019 ± 0.061	No
	HK2 (Y)	−0.011	+0.050	+0.022	+0.020 ± 0.030	No
Median RMSD	HK1 (H)	−0.266	−0.014	−0.048	−0.109 ± 0.137	Yes
	HK1 (Y)	−0.058	−0.042	−0.021	−0.040 ± 0.019	Yes
	HK2 (H)	+0.218	−0.111	+0.365	+0.157 ± 0.243	No
	HK2 (Y)	+0.011	−0.004	−0.038	−0.010 ± 0.025	No

Because the free and glucose-bound replicates are independent simulations, the pairing by replicate label is nominal; sign consistency is therefore used only as a qualitative indicator of reproducibility (Methods, Section 2.2).

Table S3. Per-replicate local free-energy well stiffness k_{loc} (mean of 400 bootstrap resamples and 95% confidence interval) and effective volumetric compliance κ_T^{eff} (full-series estimate and 95% confidence interval from a moving-block bootstrap with 1000 resamples), together with the between-replicate mean ± SD (n = 3) reported in Table 1 of the main text.

System	Replicate	k_{loc} (k_BT/nm²)	k_{loc} 95% CI	κ_T^{eff} (10^{-3} bar⁻¹)	κ_T^{eff} 95% CI
HK1 (H)	R-1	1,366	1,163–1,557	7.86	6.58–9.08
	R-2	367	269–483	25.5	22.9–28.2
	R-3	495	439–551	38.8	34.0–43.4
	**Mean ± SD**	**743 ± 543**		**24.1 ± 15.5**	
HK1 (H) +Glu	R-1	1,131	1,026–1,235	22.9	20.4–25.3
	R-2	797	766–832	134.7	121.3–147.2
	R-3	750	680–810	42.9	39.2–46.4
	**Mean ± SD**	**892 ± 208**		**66.9 ± 59.6**	
HK1 (Y)	R-1	20,599	9,080–23,597	0.935	0.847–1.013
	R-2	7,347	5,506–9,373	0.971	0.860–1.056
	R-3	7,666	5,981–9,766	0.851	0.762–0.938
	**Mean ± SD**	**11,871 ± 7,561**		**0.919 ± 0.061**	
HK1 (Y) +Glu	R-1	44,985	39,490–50,615	0.273	0.245–0.303
	R-2	11,644	8,690–14,413	0.472	0.420–0.525
	R-3	25,395	21,351–29,390	0.349	0.299–0.396
	**Mean ± SD**	**27,341 ± 16,755**		**0.365 ± 0.101**	
HK2 (H)	R-1	999	659–1,307	9.23	8.21–10.3
	R-2	826	743–907	54.3	49.4–59.4
	R-3	1,115	923–1,291	12.4	11.1–14.1
	**Mean ± SD**	**980 ± 145**		**25.3 ± 25.2**	
HK2 (H) +Glu	R-1	277	225–326	39.1	35.0–43.6
	R-2	194	104–353	46.8	38.0–54.0
	R-3	415	366–463	47.9	42.4–53.0
	**Mean ± SD**	**295 ± 112**		**44.6 ± 4.8**	
HK2 (Y)	R-1	3,003	2,235–3,707	1.48	1.31–1.65
	R-2	6,480	5,868–7,088	1.37	1.17–1.55
	R-3	4,058	3,301–4,778	1.61	1.44–1.78
	**Mean ± SD**	**4,514 ± 1,783**		**1.49 ± 0.12**	
HK2 (Y) +Glu	R-1	2,289	1,623–2,869	2.16	1.97–2.37
	R-2	4,621	3,823–5,458	1.56	1.39–1.73
	R-3	2,418	1,851–2,982	2.48	2.24–2.71
	**Mean ± SD**	**3,109 ± 1,311**		**2.07 ± 0.47**	

k_{loc} in units of k_BT/nm²; κ_T^{eff} in units of 10^{-3} bar⁻¹. Both descriptors are derived from the R_g distribution of each replicate (Methods, Sections 2.4 and 2.6).

Table S4. Quantitative metrics of glucose persistence in each binding site over the 100 ns trajectory.

Enzyme	Binding site	RMSD median (nm)	RMSD p95 (nm)	Frames in cavity <0.35 nm (%)	Mean contact occupancy	Persistent contacts (occupancy)
HK1 (H)	N-site (regulatory)	0.10	0.35	99.6	0.75	GLU260, GLU294, LYS173, ASP209, THR172, ASN208, ASN235
HK1 (H)	C-site (catalytic)	0.14	0.37	99.1	0.68	GLU708, PRO605, ASP657, ASN656, THR658
HK2 (H)	N-site	0.16	0.32	98.5	0.65	GLU260, ASP209, SER155
HK2 (H)	C-site	0.12	0.29	99.3	0.71	ASP657, GLU742, ASN683, ASN656
HK1 (Y)	single site	0.05	0.10	99.9	0.81	ASP211, SER158, THR175, LYS176, PRO160, ARG173, ASN237
HK2 (Y)	single site	0.08	0.14	99.9	0.60	ASN193, ASP194

RMSD values are protein-aligned ligand RMSD. "Frames in cavity" is the percentage of trajectory frames with ligand RMSD below 0.35 nm relative to the equilibrated pose. "Mean contact occupancy" is averaged over the pocket residues automatically detected within 4.5 Å of glucose in the equilibrated structure (Methods, Section 2.8). Residue numbers in this table follow the simulation-topology numbering, which corresponds directly to the residue indices of the deposited trajectory files (see Materials and Methods).

Table S5. Per-replicate occupancy of the dominant R_g basin and apparent residence time (ART). The basin of each enzyme is the one defined on its pooled free-state PMF (Table 2); ART is the mean duration of the continuous segments spent in the basin, computed on a common 0.1 ns grid and, for reference, at the native sampling interval.

System	Condition	Replicate	Occupancy	ART (ns), 0.1 ns grid	Segments in basin	ART (ns), native sampling	Native interval (ns)
HK1 (H)	free	R-1	0.993	19.9	5	7.09	0.01
		R-2	0.949	4.5	21	0.93	0.01
		R-3	0.883	2.8	32	0.59	0.01
	+Glu	R-1	0.993	24.9	4	3.68	0.01
		R-2	0.351	1.4	25	0.37	0.01
		R-3	0.873	2.1	42	0.40	0.01
HK1 (Y)	free	R-1	0.613	3.9	16	1.43	0.01
		R-2	0.671	2.1	32	0.88	0.01
		R-3	0.607	2.6	23	1.12	0.01
	+Glu	R-1	0.090	0.4	21	0.15	0.01
		R-2	0.003	0.2	2	0.07	0.01
		R-3	0.695	1.3	53	0.58	0.01
HK2 (H)	free	R-1	0.998	50.0	2	7.13	0.01
		R-2	0.831	2.1	40	0.55	0.01
		R-3	0.996	25.0	4	3.69	0.01
	+Glu	R-1	0.784	2.1	37	0.48	0.01
		R-2	0.719	1.6	46	0.33	0.01
		R-3	0.712	1.2	59	0.27	0.01
HK2 (Y)	free	R-1	0.935	4.3	22	0.58	0.01
		R-2	0.942	4.5	21	0.74	0.01
		R-3	0.997	16.6	6	5.25	0.01
	+Glu	R-1	0.963	3.7	26	0.62	0.01
		R-2	0.955	4.0	24	0.51	0.01
		R-3	0.948	3.8	25	0.64	0.01

The basin is the region within 2 k_BT of the minimum of the pooled free-state PMF; in HK1 (Y), its lower limit is the free-energy maximum (0.23 k_BT above the minimum) that separates it from a secondary, more compact minimum (Table 2 of the main text). ART values of replicates with few segments in the basin are bounded by the trajectory length (≈100 ns). All trajectories were saved every 0.01 ns; ART at the native interval resolves recrossings of the basin limits shorter than 0.1 ns and is therefore shorter than on the 0.1 ns grid. Replicate R-2 of glucose-bound HK1 (Y) visited the basin in only two short segments, so its ART is not informative.

Table S6. Jensen–Shannon divergence (JSD, base 2) and Bhattacharyya distance (BD) between the free and +Glu distributions of R_g and RMSD, computed with kernel-density estimates (KDE; values reported in Table 3) and with Freedman–Diaconis histograms. Pooled: pooled free-state versus pooled +Glu ensembles; cross: mean ± SD over the nine free/+Glu replicate pairs; baseline: mean ± SD over the six pairs of replicates of the same condition.

Coordinate	System	Estimator	JSD pooled	BD pooled	JSD cross	JSD baseline	BD cross	BD baseline
R_g	HK1 (H)	KDE	0.115	0.112	0.305 ± 0.190	0.377 ± 0.130	0.326 ± 0.238	0.399 ± 0.184
		Histogram	0.119	0.117	0.314 ± 0.189	0.390 ± 0.131	0.339 ± 0.240	0.418 ± 0.190
	HK1 (Y)	KDE	0.222	0.205	0.382 ± 0.235	0.274 ± 0.289	0.474 ± 0.379	0.330 ± 0.426
		Histogram	0.232	0.220	0.394 ± 0.235	0.284 ± 0.289	0.501 ± 0.397	0.348 ± 0.441
	HK2 (H)	KDE	0.115	0.097	0.251 ± 0.110	0.227 ± 0.136	0.239 ± 0.129	0.202 ± 0.131
		Histogram	0.120	0.102	0.261 ± 0.111	0.237 ± 0.139	0.253 ± 0.132	0.215 ± 0.137
	HK2 (Y)	KDE	0.139	0.111	0.296 ± 0.229	0.252 ± 0.293	0.301 ± 0.278	0.296 ± 0.419
		Histogram	0.143	0.115	0.304 ± 0.230	0.258 ± 0.295	0.314 ± 0.285	0.308 ± 0.431
RMSD	HK1 (H)	KDE	0.097	0.071	0.240 ± 0.198	0.219 ± 0.138	0.226 ± 0.214	0.196 ± 0.146
		Histogram	0.102	0.075	0.250 ± 0.197	0.230 ± 0.137	0.239 ± 0.218	0.209 ± 0.149
	HK1 (Y)	KDE	0.504	0.552	0.636 ± 0.234	0.324 ± 0.340	0.990 ± 0.630	0.463 ± 0.668
		Histogram	0.517	0.584	0.653 ± 0.226	0.347 ± 0.334	1.039 ± 0.629	0.502 ± 0.690
	HK2 (H)	KDE	0.237	0.196	0.492 ± 0.272	0.386 ± 0.349	0.662 ± 0.481	0.564 ± 0.688
		Histogram	0.246	0.207	0.499 ± 0.266	0.395 ± 0.345	0.672 ± 0.475	0.580 ± 0.696
	HK2 (Y)	KDE	0.038	0.028	0.099 ± 0.093	0.111 ± 0.086	0.081 ± 0.085	0.089 ± 0.077
		Histogram	0.041	0.031	0.105 ± 0.094	0.117 ± 0.086	0.087 ± 0.088	0.095 ± 0.078
